# Wireless Multimedia Sensor Networks: Current Trends and Future Directions

**DOI:** 10.3390/s100706662

**Published:** 2010-07-09

**Authors:** Islam T. Almalkawi, Manel Guerrero Zapata, Jamal N. Al-Karaki, Julian Morillo-Pozo

**Affiliations:** 1 Computer Architecture Department, Technical University of Catalunya, Barcelona, Spain; E-Mails: guerrero@ac.upc.edu (M.G.Z.); jmorillo@ac.upc.edu (J.M.P.); 2 Computer Engineering Department, The Hashemite University, Zarqa, Jordan; E-Mail: jkaraki@hu.edu.jo (J.N.A.-K.)

**Keywords:** wireless multimedia sensor networks, wireless sensor networks, multimedia delivery, survey

## Abstract

Wireless Multimedia Sensor Networks (WMSNs) have emerged and shifted the focus from the typical scalar wireless sensor networks to networks with multimedia devices that are capable to retrieve video, audio, images, as well as scalar sensor data. WMSNs are able to deliver multimedia content due to the availability of inexpensive CMOS cameras and microphones coupled with the significant progress in distributed signal processing and multimedia source coding techniques. In this paper, we outline the design challenges of WMSNs, give a comprehensive discussion of the proposed architectures, algorithms and protocols for the different layers of the communication protocol stack for WMSNs, and evaluate the existing WMSN hardware and testbeds. The paper will give the reader a clear view of the state of the art at all aspects of this research area, and shed the light on its main current challenges and future trends. We also hope it will foster discussions and new research ideas among its researchers.

## 1. Introduction

The field of Wireless Sensor Networks (WSNs) is receiving much attention in the networking research community and as an interdisciplinary field of interest. WSNs are becoming more low-cost, low-power, multi-functional, and viable due to the advances in micro-electro-mechanical systems (MEMS), low power and highly integrated digital electronics, and proliferation of wireless communications [1]. Wireless sensor networks (WSNs) typically consist of a large number of intelligent battery-powered sensor nodes with sensing, processing and wireless communicating capabilities [2]. The sensing circuitry measures simple ambient conditions, related to the environment surrounding the sensor such as temperature, humidity or light, and transforms them into an electric signal. Processing such a signal reveals some properties about objects located and/or events happening in the vicinity of the sensor. The sensor sends such collected data (called as scalar data), usually via radio transmitter, to a command center (sink) either directly or through multiple wireless hops [1, 3, 4]. WSNs have wide and varied applications such as real time tracking of objects, monitoring of environmental conditions, monitoring of health structures, and preparing a ubiquitous computing environment, *etc.* [1].

The above mentioned characteristics impose a lot of restrictions on the WSNs design such as fault tolerance, scalability, production costs, network topology, operating environment, hardware constraints, power consumption, *etc.* These challenges have led to an intensive research in the past few years that addresses the potential collaboration among sensors in data gathering and processing. In most applications, the deployment area has no existing infrastructure for either energy or communication. Therefore, a basic requirement for sensor nodes is to be able to survive with a limited source of energy which is usually a small battery [5]. The network should stay alive and active for a duration of time that depends on the application of the deployed network, and that may last from several weeks to a few years.

Nevertheless, the rapid development and progress of sensors, MEMS, embedded computing, in addition to the availability of inexpensive CMOS (Complementary Metal Oxide Semiconductor) cameras and microphones coupled with the significant progress in distributed signal processing and multimedia source coding techniques, allowed for the emergence of so called wireless multimedia sensor networks. As a result, Wireless Multimedia Sensor Network (WMSN) [6] is a network of wirelessly interconnected sensor nodes equipped with multimedia devices, such as cameras and microphones, and capable to retrieve video and audio streams, still images, as well as scalar sensor data. WMSNs promise a wide range of potential applications in both civilian and military areas which require visual and audio information such as surveillance sensor networks, law-enforcement reports, traffic control systems, advanced health care delivery, automated assistance to elderly telemedicine, and industrial process control. In these applications multimedia support has the potential of enhancing the level of information collected, enlarging the range of coverage, and enabling multi-resolution views [7] (*i.e.*, in comparison to the measurements of scalar data).

**Figure 1.**
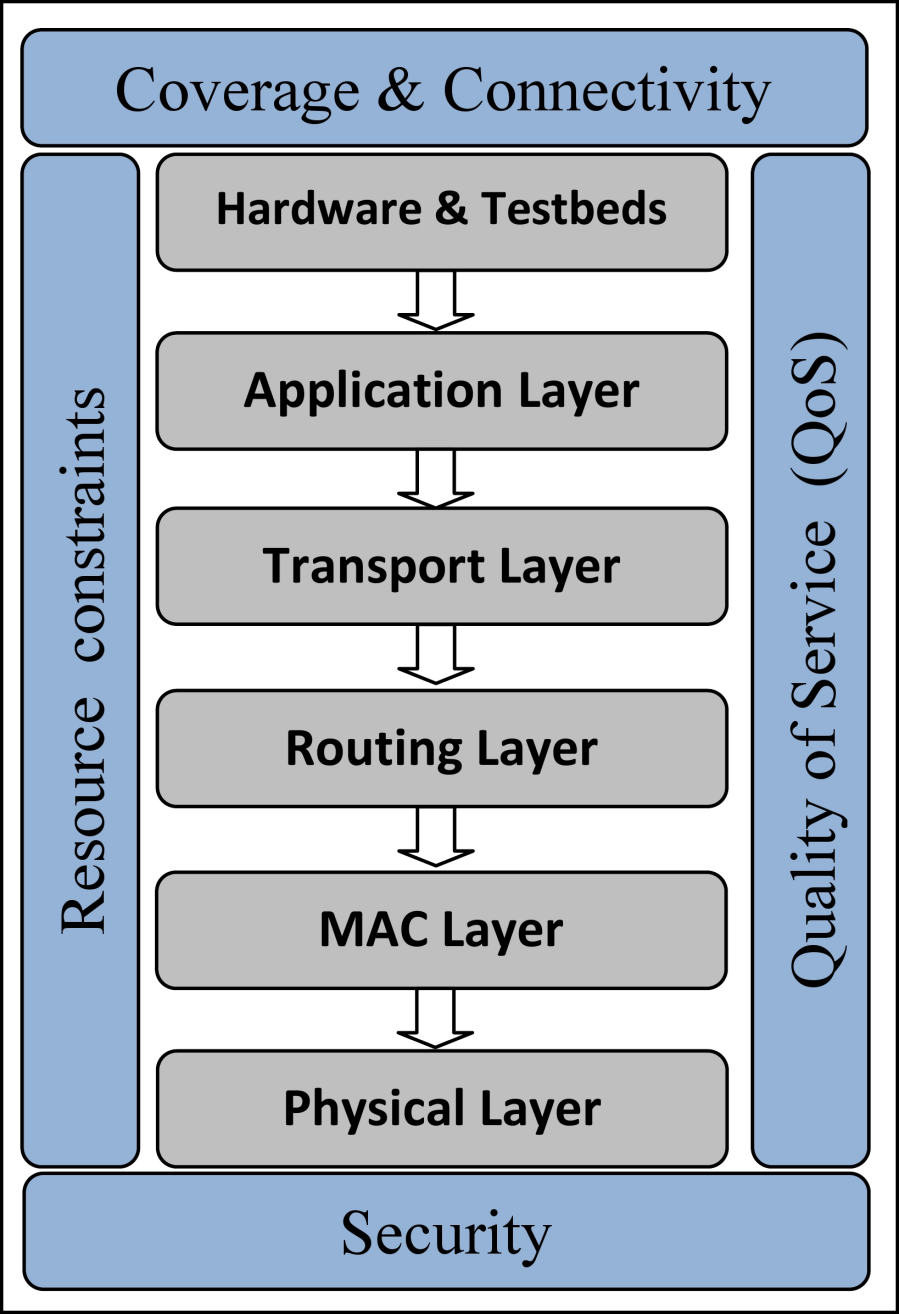
Research Challenges in WMSNs.

WMSNs have also additional characteristics and challenges, in addition to those of WSNs, because of the nature of the real time multimedia data such as high bandwidth demand, real-time delivery, tolerable end-to-end delay, and proper jitter and frame loss rate. Moreover, there are many different resource constraints in WMSNs involving energy, bandwidth, data rate, memory, buffer size and processing capability because of the physically small size of the sensors and the nature of the multimedia application that is typically producing a huge amount of data. Therefore, to meet the quality of service (QoS) requirements and to use the network scarce resources in a fair and efficient manner, these characteristics of WMSNs along with other research issues such as coverage and security —as shown in Figure 1—become a concern, and should be considered probably at the different layers of the communication protocol stack. We outline and discuss these issues in detail in the following sections. Moreover, given the relatively high redundancy in the visual sensor data, WMSNs have additional requirements such as in-node multimedia processing techniques (e.g., distributed multimedia source coding and data compression), application-specific QoS requirements, and multimedia in-network processing techniques (e.g., storage management, data fusion and aggregation).

These mentioned characteristics, challenges, and requirements of designing WMSNs open many research issues and future research directions to develop protocols, algorithms, architectures, devices, and testbeds to maximize the network lifetime while satisfying the quality of service requirements of the various applications.

In this paper, we survey the state of the art in the proposed algorithms, protocols, and hardware for the development of WMSN and discuss their open research issues. Also, we outline in detail the research challenges at different layers of the communication protocol stack. Although our survey paper and the work presented in [6] are similar in the coverage, they have many major differences. First, our paper is more up-to-date and covers all research aspects of WMSNs, (e.g., network architecture, communication layer stack, cross layer design, challenge issues like security and coverage, and hardware and testbeds). Some of those aspects were not covered in the previous work. Second, new classifications, (e.g., source coding techniques) are covered in more details. Third, a complete classification of the hardware platforms used in WMSN based on their functionalities and capabilities pertaining to wireless motes, camera motes, and testbeds is included in our paper. Moreover, we survey all the hardware devices and prototypes used in WMSN and compare among their specifications and features. More complete description and comparisons are presented in our paper, (e.g., the physical layer technologies used in WMSN, MAC layer protocols proposed for WMSN, methodologies used in designing the routing protocols in the routing layer). Finally, we tried to stress on open issues for new researchers in this field and to give a view of what we foresee are going to be the future trends.

In particular, we describe the different network architectures for WMSNs and their characteristics in Section 2. In Sections 3–7, we discuss existing solutions and open research issues at the physical, MAC, routing, transport, and application layers of the communication stack respectively. Recent works in cross-layer design are covered in Section 8. Section 9 outlines the coverage issues in WMSN and the proposed algorithms for solving them, while in Section 10 we discuss the research challenges in designing security solutions in WMSN. In Section 11, we survey and classify the existing hardware and prototype implementations for WMSN. Finally, we conclude the paper in Section 12.

## 2. Network Architecture

Traditionally, most of the proposed network architectures in scalar wireless sensor networks are based on a flat architecture of distributed homogeneous nodes, where low-power scalar sensors are in charge of performing simple tasks such as detecting scalar physical measurements. But with the emerging of WMSN and its new applications, new types of sensor nodes besides scalar sensors (such as multimedia sensors, processing hubs, storage hubs) with different capabilities and functionalities have been used. This raises the need to reconfigure the network into different architectures in a way the network can be more scalable and more efficient depending on its specific application and QoS requirements. Therefore, based on the designed network topology, the available resources in the network can be efficiently utilized and fairly distributed throughout the network, and the desired operations of the multimedia content can be handled. In general, Network architectures in WMSNs can be divided into three reference models:

The first model is the **single-tier flat architecture** where the network is deployed with homogeneous sensor nodes of the same capabilities and functionalities. In this model all the nodes can perform any function from image capturing through multimedia processing to data relaying toward the sink in multi-hop basis. Single-tier flat architecture is easy to manage. Moreover, multimedia processing is distributed among the nodes, which prolongs network life time. The second model is the **single-tier clustered architecture** deployed with heterogeneous sensors where camera, audio and scalar sensors within each cluster relay data to a cluster head. The cluster head has more resources and it is able to perform intensive data processing. The cluster head is wirelessly connected with the sink or the gateway either directly or through other cluster heads in multi-hop fashion. The third model is the **multi-tier architecture** with heterogeneous sensors. In this architecture, the first tier deployed with scalar sensors performs simple tasks, like motion detection, the second tier of camera sensors may perform more complicated tasks as object detection or object recognition, and at the third tier more powerful and high resolution camera sensors are capable to perform more complex tasks, like object tracking. Each tier may have a central hub to perform more data processing and communicate with the higher tier. The third tier is connected wirelessly with the sink or the gateway. This architecture can accomplish tasks with different needs with better balance among costs, coverage, functionality, and reliability requirements. On the other side, the use of just one node type in homogeneous flat network is not scalable enough to enclose all complexity and dynamic range of applications offered over WMSNs.

In [8], a two-tier video surveillance system of multiple cameras is integrated with a wireless sensor network mounting passive infrared (PIR) sensors to improve the accuracy of the vision system. In the first tier, PIR sensors can be used to cover the areas that are invisible to cameras or out of their FoVs (Field of Views), and to reduce the required zooming and resolution capabilities of the cameras. PIR is very suitable to WMSN because of its low cost, low power, small size, and easy to place and it is widely used for motion detection and triggering cameras. PIR’s characteristics can be exploited to detect motion and/or change in direction in its coverage area. The second tier is composed of cameras for object extraction and tracking. By using this system, changes in the motion direction can be detected by putting PIR sensors behind the obstacles and changes in background is interpreted correctly by examining the output of PIR sensor that is close to that background object; if the camera detects a moving object but the PIR sensor does not capture it, then it is considered as a change in the background.

An object detection and high resolution image acquisition via a two-tiered heterogeneous sensor network is presented in [9] that uses stereo image sensor nodes at the lower tier and high resolution actuated cameras at the upper tier of the network. The low-cost, low-power, non-actuated resource constrained stereo image sensors are used to compute the 3-D object location after detecting it in order to adjust the pan/tilt/zoom settings for the high imaging actuated platforms, Canon PTZ camera (VC-C4R). The work shows that the use of two-tiered network has a significant effect in reducing energy consumption and minimizing loss in detection of high resolution image acquisition systems comparing to single-tiered system. Two Cyclops platforms are used as stereo camera node and configured to continuously monitor the network after they finish from the process of camera calibration. If an object is detected, by using simple frame differencing, then one of the Cyclops pair will start the process of computing the 3-D location of the detected object and adjusting the associated pan/tilt/zoom settings of the high resolution actuated camera at the upper-tier. Finally, the adjusting information computed by the lower-tier platforms is transmitted to the upper tier platform which is actuated accordingly to start capturing high resolution images of the object.

A multi-tier multi-modal network, using different sensing modalities and capabilities, is presented in [10] for environmental monitoring. The network is self-organized into three tiers of distinct sensor nodes, where the first tier is equipped with passive infrared (PIR) sensors that are capable of detecting objects in the sensing field as a first task in an environmental monitoring or tracking application. The second tier is equipped with smart visual camera sensors that can identify objects in their FoV by determining the object’s predominant color. The third tier is equipped with visual sensors that are capable for computing the location of identified objects and their moving path and responsible for target tracking, as they move through the network after identifying them by the second tier sensors. During the network operation, the PIR sensors in the first tier are always active, as they are low power sensors, and monitoring the network. In case they detect any object, they alert the camera sensors in the second tier and wake them up to start capturing images for the detected objects and try to identify them. In case of successfully identifying the objects by their predominant color, the camera sensors in the second tier will wake up the powerful visual sensors in the third tier in order to track them.

In [11], an integrated mobile surveillance system of two-tiers for WMSN called (iMouse) is presented, which uses static and mobile wireless sensors for detecting and analyzing unusual events. The system consists of several static sensor nodes, some mobile sensors, and an external server or base station. The static nodes (MICAz-type nodes) form a typical WSN to monitor the environment and collect three types of data (light, sound, and temperature) and notify the base station of unusual events. The locations of these sensors are assumed to be known using GPS or any localization system. The mobile sensors (composed of Lego car and Stargate board connected with wireless mote, webcam, and WLAN card) move to event location when they are notified by the base station to capture images and transmit them to base station. If the number of detected events is less than or equal to the number of mobile nodes, the base station schedules mobile nodes to visit the emergency sites using the maximum matching technique in a bipartite graph to conserve their energy as much as possible. But if the number of detected events is more than the number of mobile nodes, the base station first clusters the emergency sites before it schedules the mobile nodes to each cluster.

## 3. Physical Layer in WMSN

The Physical Layer in WMSNs consists of the basic hardware transmission technologies of a network and defines the means of transmitting raw bits, rather than logical data packets, over the wireless link that is connecting network nodes. It is responsible also for frequency selection, modulation and channel encoding. In WMSNs, the physical layer should be designed in a way that it underlies all the higher-layer communications-related functions and meets the specific requirements and characteristics of WMSN. Therefore:

- The physical layer technology must work in a compatible way with higher layers in the protocol stack to support their application-specific requirements and to meet the design challenges of WMSN. This can be done with higher efficiency if a cross-layer model is used especially between physical layer and MAC layer.
- The physical layer should utilize the available bandwidth and data rate in the best possible way, and to be more power efficient.
- The physical layer should have a good performance (gain) against noise and interference and provide enough flexibility for both different channel and multiple path selection.
- The cost of the radio should be taken into account since it will be deployed in large number of nodes.

Physical layer technologies can be classified either into three groups (Narrow band, Spread spectrum, Ultra-Wide band (UWB) technologies) based on the modulation scheme and bandwidth consideration [12], or into different standard protocols (IEEE 802.15.4 ZigBee, IEEE 802.15.1 Bluetooth, IEEE 802.11 WiFi, 802.15.3a UWB). ZigBee [13] is the most common standard radio protocol used in wireless sensor networks because of its lightweight standard and its low-cost and low-power characteristics. ZigBee supports: data rate up to 250 kbps at 2.4 GHz, more than 65,000 nodes, coding efficiency of 76.52 %, and range of 10–100 meters. ZigBee standard is being used by most of WSN devices such as MICA-family, Tmote sky, and imote2. However, ZigBee standard is not suitable for high data rate applications such as multimedia streaming over WMSN and for guaranteeing application-specific QoS. On the other hand, other standards like Bluetooth and WiFi have higher data rate and code efficiency — as shown in Table 1— but they consume more energy. Bluetooth has been used in [14, 15] for wireless communication in WMSN, while WiFi has been used with Stargate device in many projects as shown in the Hardware section later on.

UWB [16, 17]—with coding efficiency of 97.94%, data rate up to 250 Mbps, and nominal range of 10 meters in addition to its immunity to multipath propagation and precise positioning capabilities—has the potential to enable low power consumption, high data-rate of short range wireless communication and seems to be a promising candidate for the physical layer standard of WMSN. UWB spreads the information over a large bandwidth, about 20% of the center frequency or more than 500 MHz. The physical layer of UWB is implemented by using either impulse radio (IR) of extremely short duration pulses, or multiband orthogonal frequency division multiplexing (MB-OFDM) where hybrid frequency hopping and OFDM are applied. IR-UWB has simpler transmitter and rich resolvable multipath components, but it needs a long channel acquisition time and requires high speed analog-to-digital converters, while MB-OFDM-UWB offers robustness to narrowband interference, spectral flexibility, and efficiency but it needs slightly complex transmitter. The multiple access of IR-UWB can be realized by using direct sequence UWB (DS-UWB), or time hopping UWB (TH-UWB). The low duty cycle of IR-UWB (<1%), because of the short duration of the pulse, is a key advantage for low power consumption in WMSN, also spreading information over wide bandwidth decreases the power spectral density and in turn reducing the interference with other systems and lowering the probability of interception.

For the network layer, with the new characteristics of UWB such as low power transmission and low accurate ranging capabilities, the addressing and location-aware routing protocols can be optimized for better performance and they can get rid from the overhead caused by traditional flooding technique for routing and IPv6 scheme for addressing. Also UWB properties, especially the positioning capabilities, can be exploited to simplify the hardware used in location-aware routing instead of using of GPS enabled devices. It is pointed in [16] that UWB characteristics should be taken in account in the MAC layer for channel access, scheduling, and error control wherein the low duty cycle and low power transmission reduce the probability of collisions between pulses and interference. TH-IR-UWB system could be used, for example, for simultaneous transmissions based on the adoption of different time hopping code on each active link; and the rich resolvable multipath components of IR-UWB could be exploited, for example, at the receiver side for multipath diversity and channel estimation.

It is worth to point out that, in order to further increase capacity and mitigate the impairment by fading and co-channel interference, multi-antenna systems such as antenna diversity, smart antenna, and MIMO systems, can be combined with UWB. Since UWB has almost impulse-like channel response, the combination with multiple antenna techniques such as MIMO systems may give the possibility of short-range networks with multi-gigabit rates. However, these physical-layer techniques have many challenging problems to be developed for WMSNs. Although UWB appears to be a promising alternative physical layer technology and it has many attractive features, it is still not very mature and there are many challenges and issues that need to be resolved and better understood.

## 4. MAC Layer in WMSN

The design of highly efficient and reliable medium access control (MAC) protocols is critical in wireless sensor networking. Conventionally, the goal is to provide sufficient transmission capacity at the minimum energy cost under a moderate network load condition. A quick look into the existing MAC protocols for WSNs, as surveyed in [18], reveals that lack of standardization and application-specific diverse requirements has deprived WSNs from having a single de-facto standard MAC protocol.

**Figure 2.**
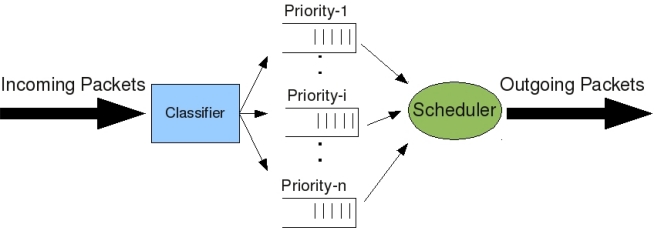
Traffic Differentiation and Priority Queueing in WMSNs.

MAC in WMSNs is essential to coordinate the channel access among competing devices. Given the energy constraints of the small, battery powered sensor devices, it is desirable that the MAC layer provides reliable, error-free data transfer with minimum retransmissions while meeting the QoS requirements ((*i.e.*, bit error rate, transmission rate, delay, fairness, *etc.*) with efficient resource utilization. MAC layer attempts to address these issues by enforcing channel access, scheduling policies (Figure 2), and error control. Therefore, a proposal of MAC layer protocol for WMSNs should satisfy the following features:

- maximize network throughput,
- enhance transmission reliability,
- minimize control overhead,
- be energy-efficient,
- and guarantee a certain level of QoS.

### 4.1. The Affecting Characteristics of WMSN on MAC Protocol Design

In WMSNs, a sensor node may have various kind of sensors, as described in the Hardware section, that gather different types of data with different levels of importance. Therefore, WMSNs generate different traffic classes and these classes require classification, buffering, and different type of services (Figure 2). In addition, a WMSN normally demands larger bandwidth and entails higher network throughput to transport large volume of data to remote data sink rapidly and reliably. However, data rates provided by existing commercial sensor products, e.g., 250 Kbps in MICAz, pose some limitations to support multimedia traffic. On the other hand, current sensor nodes, such as MICAz and WINS, already support multiple channels for communication, for example, 40 channels in WINS [19]. Thus, the development of multi-channel MAC protocols, which can effectively utilize the available channel capacity appears as a research direction to achieve a better support for multimedia applications over WSNs. Moreover, the design of the MAC layer depends on a trade-off between complexity/cost and the network throughput, which is reflected in the literature of MAC protocol design for wireless sensor networks.

The work in [19] argues that a flat architecture is not suitable for multimedia applications because the transmission of the large volume of data resulting from multimedia applications will quickly drain the battery of the sensor nodes, thus, significantly reducing the network lifetime. Of course, multimedia transmission poses a research challenge and it is true that the multi-tier paradigm is very used in the literature of WMSNs. Although they can be of interest for some scenarios, we do not think, however, that the research focus should be put on the design of MAC protocols that assume some kind of *supernodes* (*i.e.*, abundant power supply, out-of-band channel to communicate with the sink, *etc.*) that allow to get rid of the constraints of WMSNs (such as [19]).

### 4.2. MAC Layer’s Main Functions

#### Channel Access

Traditionally, MAC protocols for WSNs can be classified into contention-based and contention-free protocols, according to the methods of cooperation in listen state between neighboring nodes. Contention-free slotted access protocols suffer from synchronization problem and many energy wastes because of synchronization overhead, in addition to channel under-utilization and fixed time-slot assignments. Regarding the structure of these protocols, they use many slots to access the channel and relatively long listening time that wastes more energy. Time Division Multiple Access (TDMA) is the most common example of this class. On the other side, contention-based protocols are based on the random access to the channel. This provides more flexibility to handle different nodes densities, lower delay, and better throughput potential at varying traffic loads. Also, there are no synchronization issues, making these protocols rather simple. The down side of this relaxed, random access approach is the wasted energy due to idle listening and collisions produced with large preamble and hidden nodes problems. Carrier Sense Multiple Access (CSMA) based with, possible, Collision Avoidance (CA) and its variants are examples of this type of protocols.

Simplicity, flexibility and scalability of contention-based random access protocols make them attractive for WSNs. However, transmitting multimedia applications with strict QoS-guarantee offers significant new challenges over these energy-constrained sensor networks and makes conventional MAC protocols not suitable for WMSNs. Design of an efficient sensory MAC-protocol, satisfying QoS requirements, is one major step in end-to-end QoS provisioning over WMSNs.

Most Contention-based MAC protocols in WSNs, such as S-MAC [20] and T-MAC [21], were proposed to support single-channel architecture, as shown in Table 2. [19] argues that these protocols are not suitable for multimedia applications (because they are designed to be energy efficient at the cost of increased latency and degraded network throughput) and proposes a multi-channel MAC protocol. Of course, neither S-MAC nor T-MAC was originally thought for WMSNs and probably they would not work properly under the new requirements of such networks. We think that exploiting the multi-channel features in the existing sensor platforms is a promising direction in designing a MAC protocol for WMSN, but it is also a mistake to discard the single-channel paradigm. In fact, we will see a proposed single-channel MAC protocol for WMSNs, [22] (further developed in [23]), that clearly outperforms both T-MAC and S-MAC in terms of MAC latency (both T-MAC and S-MAC attain an average transmission delay of 60 ms, while the delay of [23] is less than 30 ms), MAC throughput (while S-MAC and T-MAC achieves a throughput of 20 Kbps and 10 Kbps, the proposal in [23] achieves an average throughput of 50 Kbps), and energy efficiency (it consumes less energy than S-MAC and 14–18% more than T-MAC).

COM-MAC is an on-demand multi-channel contention-free MAC protocol proposed in [19]. It exploits the fact that current sensor nodes, such as MICAz and WINS, already support multiple channels for communication, e.g., 40 channels in WINS, to develop a multi-channel MAC protocol in order to effectively utilize the available channel capacity through cooperative work from the other sensor nodes. In this way, a better support for high data rates demanded by multimedia applications can be achieved.

As an example of a single-channel MAC protocol, a MAC Protocol for WMSNs is presented in [22] and [23]. It argues that CSMA methods generally offer a lower delay and better throughput, especially at lower traffic loads. Thus, it is based on the CSMA/CA MAC methods to develop a QoS-based MAC protocol for WMSNs. It adaptively adjusts the contention window depending on the QoS requirements and wireless channel characteristics and dynamically adjusts its duty cycle based on the major application traffic to preserve the sensor energy without sacrificing QoS provisioning. It updates the contention window to achieve trade-off between the period wasted on the waiting for the back-off counter to expire and the collisions because of the simultaneous transmissions of more than one sensor nodes. Subsequently depending on the traffic class, it differentiates the packets into different types and updates the contention window in different amount; for higher priority traffic classes (like streaming video) the increment step size is set to be smaller than that of the lower priority while the decrement step size is set to be greater than that of the lower priority. In this way, throughput differentiation between different traffic classes can be easily controlled and adjusted by controlling this step size. As an example, authors in [23] consider a network where the mean capacity of the links is 100 Kbps and show how they can move from a scenario where the streaming video traffic achieves a throughput of 50 Kbps followed by the 40 Kbps throughput of lower priority classes, to a scenario in which streaming video achieves a throughput of 75 Kbps at the cost of 15 Kbps throughput for lower priority classes. They also demonstrate the throughput dynamics of different traffic classes in both the presence and absence of highest priority streaming video traffic: when the streaming video is on, it obtains 45 Kbps while leaving 40 Kbps to lower priority traffic classes. When it is switched off, lower priority classes obtain 80 Kbps and when it is switched on again, it quickly re-attains a higher throughput of 50 Kbps, at the cost of the reduction of lower priority traffic classes throughput to 40 Kbps. Similar conclusions are drawn regarding delay: the lower contention window of streaming video traffic gives it the lowest delay of 10 ms, when compared with 30 ms or 70 ms of lower priority traffic classes. For energy conservation, the proposed MAC protocol is trying to save energy by dynamically changing the duty cycle of the idle listening with the current traffic condition. Of course, in such a strategy, there exists a trade-off between energy consumption and delay. Results in [23] show this phenomena: for a pretty low latency of 10 ms the energy consumed by the protocol is close to 30 mWHr, while if a relatively high latency of 20–30 ms is allowed, the energy consumption reduces to less than 15 mWHr.

A cross-layer communication approach is presented in [24], between the MAC and physical layers, to provide a better QoS for WMSN applications. It is based on the Time-Hopping Impulse Radio Ultra-Wide-Band (TH-IR-UWB) transmission techniques. While [22] improves protocols like S-MAC and T-MAC but still based on CSMA/CA, [24] takes a totally different approach and discards CSMA/CA (as COM-MAC did). This architecture tries to solve the shortcomings of using CSMA/CA for the MAC layer in WSN such as the variable and uncontrollable access delays from using random timer, idle listening from using carrier sense, and increased energy consumption due to for example hidden node problem with the objective of providing QoS for WMSNs. An evaluation through simulation is performed for a network of 49 nodes, taking into account two different groups of traffic flows with different QoS demands (flows in group 1 require 100 Kbps bandwidth, 100 ms. end-to-end delay, and 0% PER while flows in group 2 have 500 kbit/s bandwidth demand, 100 ms end-to-end delay and can admit 10% PER. Results show that sources in group 1 have a throughput of exactly 100 kbit/s, while sources in group 2 show an average throughput of about 480 kbit/s, as some packets are lost. While flows in group 1 do not lose packets, flows in group 2 lose approximately 4% of the packets, which is still below the application requirement. The 0% packet loss in group 1 directly translates into a consistently higher energy consumption which is not studied in this work. Moreover, the aggregate average end-to-end delays of the two groups are well below the threshold end-to-end delay, which suggests that simulations with more restrictive conditions should be performed to better understand the behaviour of this proposal (for example, how the admission control works or how the coordination overhead affects the overall system). On the other hand, the differences in delays between flows in the same groups are very limited between different flows, which demonstrates the basic fairness of the system, and the variance of the delay is also limited. Authors in [24] argue that this shows that under normal circumstances the system leads to much more limited jitter as compared to CSMA/CA based systems. A direct comparison between the system proposed in [24] and a CSMA/CA based system (like, for example, [23]) would be, however, of great interest to verify that.

#### Scheduling and Admission Control

Scheduling, admission control, and buffer management in WMSNs is an open research issue that has attracted the research community in last years but still not really solved. As an starting example, the work in [19] makes some strong assumptions to be considered: on one hand, it uses a coordinated channel scheduling that assumes a *relatively static* network to overcome the contention overhead incurred by multi-channel MAC protocols. On the other hand, it argues that a *flat* structure is not suitable for WMSNs. The protocol operation is divided into three sessions (*Request*, *Scheduling* and *Data Transmission*) and only a heuristic is provided to calculate the scheduling to be used based on priorities. In the cluster-based MAC protocol, COM-MAC [19], a scheduled multi-channel medium access protocol is considered within each cluster where the cluster head coordinates the communication among its members in a contention free manner within both the time and frequency domains. By this way, the nodes, which are assumed to have multiple transceivers being able to operate on a set of available channels simultaneously, avoid collision, idle listening and overhearing problems. The cluster head dynamically allocates time slots and channels for its members according to the current QoS requirements and network traffic status.

One work that attacks the scheduling and buffer management problem is [28] based on the fact that different network applications need different QoS requirements such as packet delay, packet loss, bandwidth and availability. And this can be done not by increasing network capacity (as COM-MAC [19]), but by developing a network architecture which is able to guarantee QoS requirements for high priority traffic. It argues that the sensor networks should be willing to spend more resources in disseminating packets that carry more important information by using a differentiated service model for WMSNs. The proposed model can support two major different types of traffic classes: real time class (Expedited Forwarding or EF) and non real time traffic (Assured Forwarding or AF) which is divided into three classes: high priority AF1, medium priority AF2, and low priority AF3. In this model, real time traffic is buffered in a separate queue with low buffer size while non real time traffics are managed by using random early detection (RED) queue management in separate queues also. The delay performance of different traffic classes is evaluated by using two scheduling mechanisms: Priority Queuing (PQ) and Weighted Round Robin (WRR). It is shown that by using priority queuing (PQ) scheduling mechanism for EF traffic class and weighted round robin (WRR) scheduler for non real time traffic classes, low delay bound and guaranteed network bandwidth for high priority real time traffic can be provided. This work, as it simply considers a scheduling system, does not provide any insight on the physical and MAC layers of WMSNs. The main drawback of this work, however, is that it demonstrates that the proposed system can provide differentiated services but it does not study signaling overheads or energy consumption.

In concordance with [24], the work in [16] shows also that UWB characteristics should be taken into account in the MAC layer for channel access, scheduling, and error control wherein the low duty cycle and low power transmission reduce the probability of collisions between pulses and interference.

A theoretic work done in [30] tries to give insights in where and how to deploy sensor nodes (and how many of them) so that all the nodes can be supported by the limited communication resources in WMSNs. It assumes low-mobility or a static network, flat topology, and a single-channel TDMA-based communication to present a cross-layer design model where node admission in WMSNs and its interaction with resource management and link scheduling is investigated. The interaction is formulated as a two-stage optimization problem: first stage is to maximize the number of admitted sensor nodes, and the second stage is to maximize the network life time. It is shown also that this optimization problem can be presented in equivalent one-stage optimization problem with more compact mathematical form. Note that a common characteristic in all the presented proposals is that some kind of service differentiation (Figure 2) is provided: it seems clear that without this feature it is not possible to guarantee the QoS needed by WMSNs.

#### Error Control

Due to the unreliability of the wireless medium in WMSNs, the transmitted data such as multimedia content is exposed to losses or errors mostly caused by multi-path fading, co-channel interference, jamming … *etc.* Therefore, in order to improve the perceptual quality of the received multimedia content, techniques for error correction and loss recovery should be employed in WMSNs to combat the unreliability of the wireless channel at the physical and MAC layer. Forward Error Correction (FEC) and Automatic Repeat Request (ARQ) are examples of these techniques. The usefulness of ARQ in sensor network applications is limited by the additional re-transmission cost and overhead. On the other hand, decoding complexity is greater in FEC, as error correction capabilities need to be built-in. Considering this, simple error control codes with low-complexity encoding and decoding might present the best solution for sensor networks. In the design of such a scheme, it is important to have good knowledge of the channel characteristics and implementation techniques.

A comparison between two techniques of error compensation of transmission distortion in multipath WMSN is conducted in [31]. First technique is the Error Concealment (EC) algorithm that reconstructs the distorted multimedia data as closely as the original one by utilizing the use of modified discrete wavelet transform for embedding downsized replicas of original image into itself. EC does not need increasing in bandwidth demand as well as retransmissions and consequent delay, however EC algorithm needs more processing power at the source and sink nodes comparing to the other technique. The second technique is the Forward Error Correction (FEC) based on Reed-Solomon coding that compensates and corrects the wireless link errors by utilizing the use of redundant data. FEC technique can mitigate the wireless link impairments but it cannot solve the errors from instant node failure problem and require considerable increase in the transmission bandwidth. It is shown in [31] that EC technique with multipath routing is more promising than FEC based RS coding to compensate for error and losses in WMSN.

### 4.3. Research Issues

In this section we presented a set of representative research efforts regarding MAC for WMSNs: single-channel/multi-channel and/or scheduled/contention-based proposals appear in the literature. In our opinion, multi-channel MAC protocols are more suitable for WMSNs. We have also identified different considered topologies. Although it can be interesting to exploit to some extend the use of multi-tier WMSNs in the design of MAC protocols, this should not be used to simplify the problems that the transmission of multimedia content represents. In this sense, papers that consider a flat topology are more research challenging.

We discussed the cross-layer design dependencies between MAC layer and other layers of the communication stack especially the physical layer, in the case of using UWB technology. The adoption of the UWB technology as the underlying transmission technique in WMSNs and the potential challenges in this area, appear as an interesting research topic. We believe that this research area will attract the attention of many researchers and boost the applicability of UWB in multimedia networking.

In overall, we think that cross-layering is essential for efficient MAC designs in WMSNs, together with queue-management and traffic classification/prioritization as long as QoS is required for multimedia traffic.

## 5. Routing Layer in WMSN

Routing layer in wireless sensor network aims to deliver the sensed data from the sources to the sink node taking into account several design considerations, such as energy efficiency, link quality, fault tolerance, and scalability. Although there are many routing protocols proposed for the traditional WSN, the design of routing protocols for WMSN is still an active research area. We believe that the new characteristics and constraints due to the multimedia content handling over the network make the proposed routing protocols for WSNs not directly applicable for WMSNs. The multimedia nature of the collected information (video streaming, still images, audio) adds more constraints on the design of the routing protocols in order to meet the application-specific QoS requirements and network conditions.

There are many traffic classes in WMSNs and can be categorized into three main classes or services depending on their QoS requirements: 1) Event-driven service which is delay intolerant and error intolerant but it requires less bandwidth, so a path with a little traffic and high signal to noise ratio is attractive for this kind of service. 2) Data query service is error intolerant but query-specific delay tolerant applications, so a path with significant congestion and a high signal to noise ratio may be used for this service. 3) Stream query service which is delay intolerant but query-specific error tolerant application (in a sense packet losses can be tolerated to a certain extent), so a path with less traffic and relatively lower signal to noise ratio is better for this type of service.

### 5.1. Routing Methodologies in WMSN

The recent work in routing layer of WMSN, as shown in Table 3 and summarized below, tries to handle these new characteristics of WMSNs and their design challenges by either modifying the previous work done in WSNs (e.g., using multiple performance metrics to meet the additional QoS requirements), or proposing new solutions based on new methodologies (e.g., using multi radio or MIMO systems, switching between multiple channels, selecting multi routing paths, or mixing among these methods). Moreover, we should point to the fact that these additional challenges and requirements of WMSNs, mainly by streaming real-time multimedia content, open the call for new research on cross layer design for more optimizing solutions. For example, cross layer design between multimedia source coding techniques at the application layer and the routing protocol in the routing layer can be exploited for better multipath selection or in-network processing. Also, cross layer design between the routing layer and the MAC layer can allow for packet-level service differentiation or priority-based scheduling and for more power efficient routing mechanisms.

The work in [32] presents an Ant-based Service-aware routing algorithm (ASAR), a QoS routing model for WMSN, that chooses appropriate paths for different QoS requirements from different types of services. The proposed algorithm mainly addresses the routing scheme between the cluster head and sink node in which a cluster head transfers the different classes of data. The process starts at the cluster head when it generates the ants for each type of service and then depending on the objective function of each type of data and pheromone value of each path, different paths are found to meet the different QoS requirements. In order to quicken the convergence of the algorithm and optimize network resources, the algorithm quantifies the pheromone value on the sink to decrease the sending frequency of reverse ants or control messages. Presented results show that: ASAR has a significant advantage over Dijkstra and Directional Diffusion (DD) in most metrics; except in packet loss rate for stream query service and delay when compared with DD.

A geographic routing algorithm, Two-Phase Greedy Forwarding (TPGF), is proposed in [33]. It explores near shortest hole-bypassing node-disjoint routing paths for WMSNs. TPGF supports multipath transmission by repeatedly executing the algorithm to find more on-demand node-disjoint routing paths. TPGF also supports near shortest and hole-bypassing path without including the face routing or planarization algorithms in order to maximize the number of available paths. It assumes that all the nodes know their location information and the source nodes know also the location of the base station. The first phase of TPGF is responsible for finding the possible routing path and it consists of two steps: greedy forwarding and step back & mark. The process starts at a source node that chooses the next-hop node which is closest to the sink among all the neighboring nodes and so on. In case that the next-hop does not have any farther neighbor except the previous node, it steps back to its previous-hop and marks itself as a block node. The second phase of TPGF is responsible for optimizing the found routing paths with the least number of hops and by eliminating the paths circles (if any) in the found paths using label based optimization technique. Presented results show that the average path length obtained by TPGF is much shorter than the one obtained by GPSR [34].

The work in [35] suggested to use landmark ad hoc routing protocol (LANMAR) in WMSNs with deploying limited number of mobile swarms, in which the network is divided into groups (LANMAR groups) and each group has a landmark node which is dynamically elected. A swarm is a group of nodes physically close to each other and usually share the same mobility pattern. Comparing to other sensor nodes, the swarm nodes have better capabilities in terms of hardware functionalities and networking capabilities (such as high quality video camera, multiple long radio range, large channel bandwidth, and maybe ability to communicate with satellites) and they can move with relatively high speed. An example of mobile swarm can be a group of tanks or unmanned aerial vehicles (UAV) moving together. The mobile swarms can communicate and exchange information between each other by using satellite communication or mobile backbone network (MBN). With the help of the limited number of mobile swarms, high quality of multimedia streams can be supported in large-scale sensor network. Once there is a hot or interested spot, a swarm can be directed to that area to help forwarding high quality multimedia streams. Results presented in this work show that the delivery rate and average end-to-end delay of their proposed protocol (Swarm-based LANMAR) outperforms LANMAR and AODV.

A non interfering disjoint multipath routing for WMSN is proposed in [36]. It addressed the problem of interference between multiple paths using one channel in WMSN and suggested an incremental on-demand approach in which only one path is built for a given source and additional paths are built when required in case of path congestion or lack of bandwidth. In order to solve the problem of interference between close paths, the proposed solution forces the multipath routing to build paths that are not interfering with each other from the beginning by putting all the interfering nodes of a given path in a passive state. Passive nodes do not further participate in building any other path in future and consequently will not interfere with previously built paths. The process starts at the sink when it floods the network with requests until they reach the source nodes. The source node starts immediately sending data on the selected paths and all the intermediate nodes between the source and the sink will inform their neighbors to switch to the passive state. The proposed work argues that putting some nodes in a passive or sleep mode increases the overall throughput and reduces the consumed amount of energy in the network. Results obtained through simulation show that the proposed protocol achieves better throughput with less energy consumption by using fewer non-interfering paths when compared to multipath schemes without interference awareness.

Modifications on Direct Diffusion, the routing protocol for WSN, are done in [37] to support multipath routing for WMSN based on link quality and latency metrics. One of the modifications includes using Costp, which is a product of expected transmission count (ETX) and delay, as a performance metric instead of pure delay that was used in Direct Diffusion. Since close paths interfere with each other and consequently have poor SNRs which are indirectly used to estimate ETX values, they have less probability to be selected by using Costp metric and this also will lead to increase throughput. The other modification is to reinforcing multiple links at the sink to obtain disjoint path from the source, and to match multipath routing. However, this routing protocol does not consider the bandwidth as QoS metric for routing decision or prioritizes the incoming packets to schedule them but it does consider the playout deadline in a sense that the data arrives after the deadline will be discarded. Results, show that the presented protocol for multipath video streaming over WSNs obtains higher throughput (even the double in some conditions) than its single path counterpart (EDGE) through the use of multiple disjoint paths.

A design of QoS aware routing protocol is presented in [38] to support high data rate for WMSNs by ensuring bandwidth and end-to-end delay requirements of real time data and maximized throughput of non-real time data. The routing protocol uses multiple paths, multiple channels, and QoS packet scheduling technique based on the dynamic bandwidth adjustment and path-length-based proportional delay differentiation (PPDD) techniques to meet the bandwidth and delay requirements respectively. These requirements (bandwidth and delay) are adjusted locally at each node based on the path-length and incoming traffic in static flat wireless network where all the nodes are homogeneous multimedia sensor nodes capable of performing all possible application tasks (video, audio, scalar data) and equipped with single radio interface and multi-channels. QoS-aware packet scheduling policy considers different priorities for real time packets and non-real time packets by using at each node a classifier that checks the type of the incoming packets and sends to appropriate queues, and a scheduler that schedules the packets according to the delay and bandwidth requirements. The proposed protocol -as shown through simulation-clearly improves average delay per real-time packet, average lifetime of a node, and throughput of non-real-time data when compared with single-r and multi-r mechanisms [39].

An extension for a routing protocol, multimedia enabled Improved Adaptive Routing (M-IAR), is done in [40] to enable handling multimedia content by taking into account two extra QoS parameters, end-to-end delay and jitter. M-IAR is a flat multi-hop routing protocol that exploits the geographical location of the sensor nodes, by assuming that all the nodes know their positions and the positions of their neighbors and the sink node, in order to find the shortest route containing the least number of nodes between the source and the sink. M-IAR uses two types of ants: forward ant and backward ant. Forward ant is used by the source node to explore the path toward the sink and selects the next-hop neighbor with the highest probability according to the mentioned metrics. The backward ant is used by the sink node and uses the global information from the forward ant to update the probability values and reinforce the visited nodes. Unfortunately, the authors of the paper do not compare the results obtained from the proposed protocol with any other protocol.

The work in [41] uses the game theory and ant colony algorithm to solve the problem of QoS routing in WMSN. The idea of using game theory together with the ant colony algorithm is to overcome the shortcoming of the current ant-based routing protocols for WMSNs such as: the long time needed by forward ants to find the destination and the overhead occurred from using plenty of backward ants to update the routing probability distribution. Therefore, game theory is used depending on the assumption that the sensor nodes are rational and have selfish action (*i.e.*, they try maximize their payoffs with minimum cost). Unfortunately, the paper does not obtain any kind of performance nor does compare the proposed protocol with any other protocol.

Multimedia-aware Multipath Multi-Speed (Multimedia-aware MMSPEED) routing protocol is proposed in [42]. Multimedia-aware MMSPEED is an extension over MMSPEED routing protocol to take into account the embedded information in the received packets in which near optimum path is reserved for I-packets and marginal paths are used for P-frames. MMSPEED protocol [43], which is also an extension for SPEED protocol [44] that was designed for WSN, can differentiate between flows with different delay and reliability requirements and has significant potential in video transmission applications. However, experimental results in [42] show that MMSPEED is not compatible with some special features of Multimedia traffic such as high video frame rate and packet’s information dependency.

QoS-based energy-efficient routing protocol is proposed in [45] called QuESt that is capable of carrying multimedia content over WMSNs. The proposed routing protocol aims to build set of non-dominated paths that satisfy application-specific QoS parameters based on using multi-objective genetic algorithm (MOGA). It is shown that determining optimal routes satisfying multiple QoS parameters (end-to-end delay and bandwidth) simultaneously in energy-constraint sensor network is a NP-complete problem, hence the authors prefer to use MOGA algorithm as a tool to solve this NP-problem by treating the multiple QoS parameters independently without combining them into a single objective function. The end-to-end delay is modeled by using Weibullian distribution to capture the inherent long-range dependency of data traffic, and the bandwidth is modeled by taking the product distribution of the individual links. It is assumed to have one single sink and multiple sources in the network, then the possible routing paths between the sources and the sink are found using depth first search (DFS). These initial paths then are fed to the MOGA algorithm to give a status (fitness value) for the QoS parameters for each path. Finally, the proposed routing protocol will select the path that is suitable of each type of data traffic based on the QoS parameters status.

### 5.2. Future Research Directions

In general (as we believe), protocols inspired in ant colonies and game theory will keep being published. They will look good on paper, specially with all those formulas. Nevertheless, nobody will consider seriously to put them into real use because deep down everybody will suspect that less glamorous but more down to earth approaches are the way to go. In addition, many of those protocols have a too long adaptation time to routing topology changes to be feasible to many scenarios.

Multipath routing is the way to go in WMSNs because these wireless networks need to exploit the network bandwidth to its limit and sometimes in short bursts. Of course, that makes everything more complicated, affects transport layer, and introduces many new problems. Nevertheless, we foresee this to be the approach that will win in the long run. But, the future proposals will have to be designed as integral solutions that cover routing, transport layer, security and sometimes even MAC layer.

Geographical routing has been touted by many as the routing that suits best WMSNs [46–48]. Nevertheless, we foresee exactly the opposite. This is due to the fact that these mechanisms require that nodes know their geographical positions (most of the times assuming they have a positioning system like GPS) being arguably an unfeasible requirement for many scenarios. In addition, these routing protocols have no security provisions at all. Thus, very simple attacks can be devised against them. Moreover, in WMSNs bandwidth is such a big issue, that more complex routing protocols that discover better routes are going to be required.

Quality of Service is clearly a needed feature in many WMSNs, specially if there is some sort of real time streaming. Nevertheless, there will be many scenarios in which the use of different message priorities are going to give more or less the same performance with less complexity. Expect that sooner or later the general recommendation in this issue will be: If you do not really need QoS, use message priorities.

## 6. Transport Layer in WMSN

Transport layer is a group of protocols that run over the network layer to enable end-to-end message transmission. Transport layer aims to provide several services such as: same order delivery, data reliability and loss recovery, flow and congestion control, and possibly QoS requirements (e.g., fairness and timing). TCP and UDP are examples of standard transport protocol that are currently used for the Internet. However, these traditional transport protocols cannot be directly implemented over wireless sensor network [6, 49] because WSN in general and WMSN in particular have their distinctive features, which make them different than typical Internet network, and they have very wide range of applications that need special requirements. Some of the features of WMSN are the following:

- *Network topology*: The network topology of WMSN is dynamic due to wireless link condition and node status, and generally it takes the shape of multihop many-to-one (like a star-tree) topology that is either flat or hierarchal. These variations in network topology should be taken into account in designing a transport protocol for WMSN.
- *Traffic characteristics*: Most of the traffic in WMSN is generated from the source nodes toward the sink and, depending on the application, this traffic can be continues, event-driven, query-driven, or hybrid. Also in many cases, the source node can send its multimedia traffic using multipath route to the sink and this feature can be exploited to design a suitable transport protocol for keeping the quality of multimedia streaming.
- *Resource constrains*: The sensor nodes have limited resources in terms of battery power, communication bandwidth, and memory that require less expensive and more energy efficient solutions for congestion control and reliability.
- *Application-specific QoS*: As we mentioned before, WMSN has diverse applications from surveillance and target tracking to environmental and industrial applications. These applications may focus on different sensory data (scalar, snapshot, or streaming) and therefore they need different QoS requirements in terms of reliability level, real-time delivery, certain data rate, fairness, *etc*.
- *Data redundancy*: Collected sensory data -in general-in WMSN has relatively high redundancy and hence many WMSN applications use multimedia processing, such as feature extraction, data compression, data fusion, and aggregation to decrease the amount of data while keeping the important information. Therefore, reliability against packet loss becomes an issue in WMSN especially if these packets contain important original data such as Region of Interest (ROI).

UDP (User Datagram Protocol) uses a simple transmission model without implicit hand-shaking mechanism to provide timeliness for real-time applications like streaming media, but it does not guarantee data reliability, nor does it provide flow and congestion control. On the other hand, TCP (Transmission Control Protocol) is connection-oriented transport protocol based on 3-way hand-shaking mechanism to provide reliable and ordered delivery of data. However, TCP has several disadvantages with respect to WMSN, which are:

- Overhead of the connection establishment mechanism in TCP might not be suitable for event-driven applications.
- TCP uses end-to-end congestion control that requires longer response time (comparing with hop-by-hop control) and may cause more packet loss in case of congestion.
- The reliability mechanism in TCP is also based on end-to-end retransmission which consumes more energy and bandwidth than hop-by-hop retransmission.
- TCP assumes that packet loss is due to congestion only and hence triggers the rate adjustment process to reduce the traffic rate whenever it detects packet loss. This behavior in TCP leads to decrease the throughput in WMSN because congestion is not only the reason for packet loss, also wireless link condition and bit-error level cause packet loss that cannot be solved by rate reduction.
- Fairness is an issue in TCP, because congestion control mechanism in TCP can discriminate against sensor nodes that are far away from the sink node.

### 6.1. Congestion Control

Congestion control is one of the services done by transport layer protocols to mitigate congestion in the network. Congestion, in wireless sensor networks, not only wastes the scarce energy due to a large number of retransmissions and packet drops, but also hinders the event detection reliability and link utilization. As we pointed before, there are two main reasons for causing congestion in WMSN: The first one is called node-level congestion that is due to the packet arrival rate exceeds the packet-service rate causing buffer overflow in the node and can result in packet loss, and increasing queuing delay. This is more likely happen at sensor nodes close to the sink, as they usually carry more combined upstream traffic. The second one is link-level congestion that is related to the wireless channel condition due to contention, interference, and bit-error rate. Congestion control mechanism consists of three steps: congestion detection, congestion notification, and rate adjustment. There are two ways to detect congestion either using active method such as timer or acknowledgment, or proactive method using queue length as in QCCP-PS [50], LRCC [51], CODA [52], packet service time as in CCF [53], or ratio of packet service time over packet inter-arrival time as in PCCP [54]. Congestion notification can be done either explicitly by using special control messages as in LRCC [51], or implicitly by piggybacking congestion information in normal data packets (e.g., using congestion notification, CN, bit as in QCCP-PS [50], Fusion [55], CCF [53]). Rate adjustment is done by step-by-step decreasing of the traffic rate into the congested area in case of using CN bit as in QCCP-PS [50], or using accurate rate adjustment if there is enough available information as in PCCP [54]. Some techniques as in LRCC [51] try to reduce the traffic rate into the congested area, but in the same time, avoid reducing the source stream traffic rate -in order to maintain the received quality of multimedia streaming-by balancing the stream traffic over multiple paths and reducing the traffic toward the current congested path.

A Queue based Congestion Control Protocol with Priority Support (QCCP-PS) is presented in [50] to deal with congestion control in the transport layer. QCCP-PS focuses only on congestion control as it is very important for WMSN to support different applications. QCCP-PS congestion control mechanism is based on hop-by-hop approach and consists of three parts: congestion detection unit, congestion notification unit, and rate adjustment unit. To detect congestion, QCCP-PS uses the queue length as an indication of congestion degree where there is separate queue to store input packets from each child node in addition to a queue for the source traffic from the receiving node itself. The output of this detection process is called congestion index. QCCP-PS assumes that each sensor node has different priority, and depending on that the rate assignment to each traffic source, as well as its local traffic source, is based on its priority index and its congestion index. Therefore, the sending rate of each traffic source is increased or decreased depending on its congestion degree and its priority. Congestion notification and the new adjusted rates are sent implicitly by piggybacking the new rate value of each child node with the sending data of each sensor node. It has been shown that QCCP-PS has better performance than other protocols such as Priority based Congestion Control Protocol (PCCP) that uses priority index for decreasing rate only in case of congestion.

Another congestion control framework for WMSN is proposed in [51] to provide the necessary bandwidth and to alleviate the congestion problem to multimedia streaming. The proposed congestion control mechanism is implemented on the top of multipath routing facility. By exploiting this feature, the congestion control mechanism is based on load repartitioning over the multiple paths, instead of decreasing the transmission rate in case of congestion in order to maintain the quality of video streaming. The load repartition based congestion control (LRCC) uses queue length as congestion detection indicator along with collision rate. LRCC also uses explicit congestion notification by using especial control messages (called congestion notification messages). In reception of these notification messages, the source node will try to balance its traffic on the available paths while reducing the amount of data sent on the current congested path in order to reduce the congestion as well as maintain its sending rate unchanged.

### 6.2. Reliability and Loss Recovery

Loss recovery is another service that can be done by the transport protocols and aims to insure reliable packet delivery by retransmitting the lost packets. Loss recovery mechanism consists of three steps: loss detection, loss notification, and retransmission recovery, and these steps can be done either using end-to-end as in STCP [56] or hop-by-hop as in RMST [57] approach. In many applications of WMSN, hop-by-hop approach is preferred because it is more energy efficient (no need to send control messages or data packets over multiple hops), takes less response time, and requires less memory to cache packets for recovery. However, hop-by-hop loss recovery requires intermediate nodes to cache packets for future retransmission, and cannot guarantee packet delivery in case of node failure. Loss detection is either based on the sender by using timer or overhearing, or based on the receiver by using packet sequence number as in RSTP [58]. Loss notification is done explicitly by using acknowledgment messages or implicitly by overhearing success transmission from the next hop. Retransmission in WMSN is preferred to be based on hop-by-hop approach (it is also referred as link-by-link) where the packets can be cached at the intermediate nodes for less duration and for faster retransmission in case of packet loss. However, there is an overhead of using the limited buffer space at the intermediate node for caching packets for other nodes, as well as performing timely storage and flushing operations on the buffer. Therefore, this rises the need for in-network storage mechanism [59] or collaboration-based distributed cache points over the network [60]. Some mechanisms as in DWT-Reliable [61] and MMDR [62] provide reliability for multimedia streaming transmission in WMSN by exploiting the source video coding techniques by which the source traffic can be splitted into multiple streams. Each stream can be given different priority, depending on its resolution-level of the original content, and transferred over the network using priority-based routing or multipath routes.

The same concept of using multimedia processing to prioritize data packets for reliable transmission has been used in [63] where WMSN transparent protocol, called Reliable Synchronous Transport Protocol (RSTP) is demonstrated. RSTP aims to reduce the large transmission delay of transferring multiple images between the source nodes and the sink due to transmission errors and limited bandwidth, and to provide ordered delivery. RSTP exploits the semantic of Progressive JPEG image stream to prioritize different parts of the stream and schedule the transmission based on the importance of the information. Progressive JPEG is used here rather than baseline JPEG because the first uses the coarse-to-clear data presentation mode which separates the DCT coefficients using multiple scans. By this way, the image can be divided into a number of scans that has smaller size than the original image and the low-quality (coarse) version of the image can be transmitted faster over WMSN. In RSTP, the less important parts within a data stream, which are the high frequency parts or the high quality version, would be discarded if there is not enough bandwidth. As a result, the limited bandwidth will be shared equally among the sensor nodes and the same transmission rate is maintained. Also the packets of images taken at earlier time would be scheduled first so that the temporal properties of the reference images are reserved. Once all the reference images reach the same quality level at the sink side, the image reconstruction process can start. For connection establishment and termination, RSTP uses similar technique of three-way handshake of the classic TCP protocol with the except that the receiver, not the sender, initiates the connection. For loss recovery, the receiver tracks the sequence number of the received packets and sends retransmission request if there is a gap in the sequence numbers or when the timeout alarms without receiving the last packet. RSTP incorporates TCP-ELN for the congestion control that uses transmission requests for packet losses caused by bit-errors and the classical TCP’s congestion control mechanism for packet losses due to congestion.

A transmission scheme is proposed in [61] for reliable transmission of images in wireless multimedia sensor network, which is based on two-dimensional discrete wavelet image transform (2D DWT) and semi-reliable transmission to achieve energy conservation. DWT divides the image into separable sub-bands of multi-resolution representations. For example, the image can be decomposed into 4 sub-bands (LL, LH, HL, and HH) where the LL sub-band has the half size version of the input image and contains the low-pass information, and the others contain high-pass information. The LL sub-band can also be transformed again to have more levels of resolution. Then each sub-band is compressed to reduce its size by using entropy coding for lossless compression (Lempel-Ziv-Welch, LZW is used here) because lossless compression is less complex and require less computation. As a result, image data can be divided into multiple packets of different priorities where lowest resolution image data packet has the highest priority and should be reliably transmitted. Other packets can be handled with a semi-reliable transmission policy in order to save energy. Semi-reliable transmission enables priority-based packet discarding by intermediate nodes according to their battery’s energy state. Packets of certain priorities are only forwarded by intermediate nodes if their battery energy level is above a given threshold.

Another framework that is based on multipath routing scheme is presented in [62], but this work considers offering reliability against bit-error from the wireless links only. The multipath multi-stream distributed reliability (MMDR) framework is proposed to reliably transport video content over WMSN by exploiting the features of multi-stream coding of video data and multipath routing. MMDR partitions the source encoded video data into multiple streams using one of the source coding techniques such as Layered Coding (LC), Multiple Description Coding (MDC), or Distributed Video Coding (DVC). Then Low Density Parity Check (LDPC) codes are used for channel coding the multiple video streams to compensate for the error prone wireless links. After that, MMDT will balance the video streams traffic over the available multipath route. Then at intermediate nodes, MMDR uses progressive error recovery algorithm (D-PERA) that tries to recover the bit errors by partially decoding the LDPC encoded packets.

### 6.3. Open Research Issues

**Figure 3.**
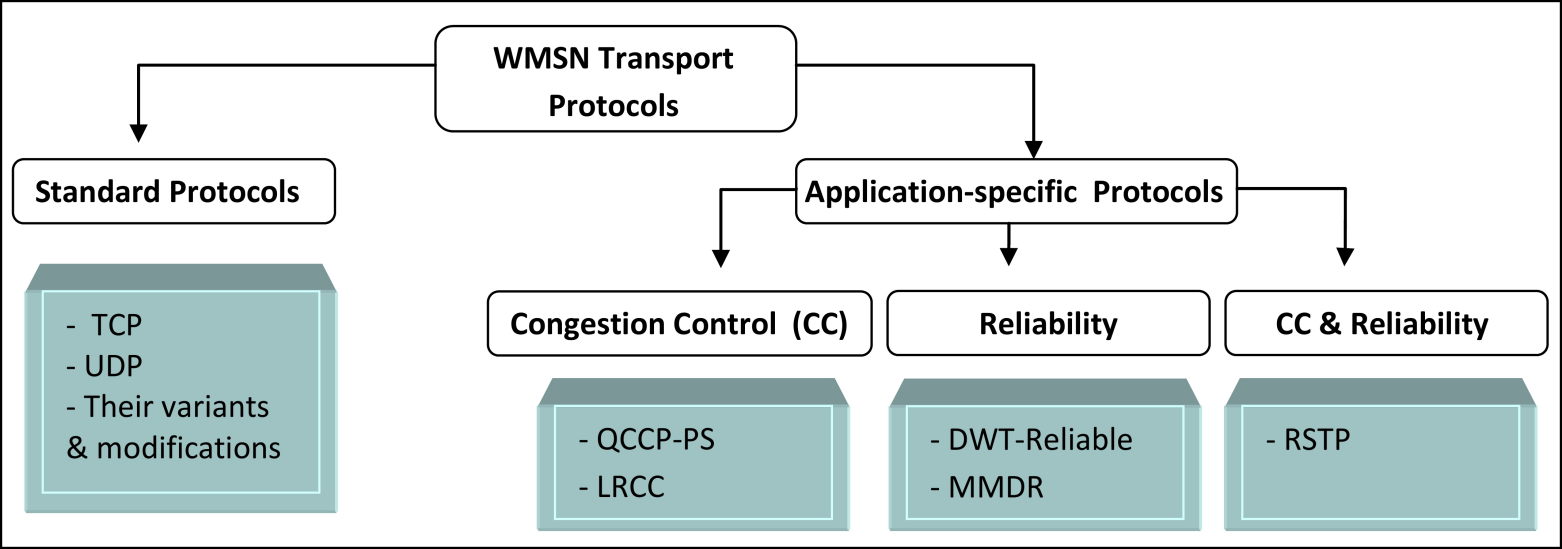
WMSN Transport Protocols Classification.

¿From the above discussion, we can conclude that the existing transport protocols in sensor networks can be classified into two groups, as shown in Figure 3: Standard protocols (which include TCP, UDP, and their variants and modifications), and Non-standard or Application-specific WMSN transport protocols (which can focus on Congestion control only, Reliability only, or both Congestion control and Reliability).

In many real-time applications, if a standard transport protocol is to be used, UDP is preferred over TCP especially when timeliness is concerned than reliability. However, because of the unique characteristics of WMSNs that we mentioned in the beginning of this section, we believe that TCP with some modifications can better handle multimedia content over WMSNs. Some work is done in this direction such as Sensor Transmission Control Protocol (STCP) [56] that proposes some modifications in the TCP header fields in order to support video transmission and differentiated service model.

Most of the proposed application-specific transport protocols do not take into consideration the multimedia requirements in WMSN and none of them addresses its diverse concerns. This can be seen clearly in the performance evaluation conducted in [64], where it was shown that many of the proposed transport protocols cannot provide acceptable video transmission and do not support real-time communication in WMSN. Therefore, we believe that designing a transport protocol with appropriate performance metrics for both reliability and congestion control and based on the application layer source coding techniques will be a promising direction in this research area.

## 7. Application Layer in WMSN

The application layer in WMSN provides heterogeneous functionalities and supports many services, which include: 1) Multimedia processing and source coding techniques that depend on the application-specific requirements and capability of the hardware. 2) Effective communication with other application programs over the network to support collaborative in-network multimedia processing mechanisms. 3) Traffic Management and Admission Control.

### 7.1. Multimedia Source Coding Techniques

In order to have the ability to handle multimedia content over wireless sensor networks and to support real time multimedia applications, multimedia processing and source coding techniques have been widely used in the application layer of WMSN. Multimedia processing techniques aim to reduce the amount of multimedia traffic transferred over the network by extracting the useful information from the captured images and videos while in the same time maintaining the application-specific QoS requirements. However, in WMSN, these techniques should be designed in such a way that they meet current hardware capabilities, more power efficient to match the battery constrains in WMSN, and have high compression efficiency to reduce the size of the multimedia content and to meet the available supported data rate and bandwidth in the network.

The existing video coding techniques that have been used in the literature for WMSN vary in their high compression efficiency, error resiliency, and low encoding-decoding complexity and can be classified into four groups:

- Layered Coding (LC) [65] is type of video source coding technique by which the original data is encoded to one important base layer (coarse version) and one or more less important successive enhancement layers (to get the fine version). At the destination side, the base layer can be combined again with all or subset of the higher-quality layers to achieve the desired level of video resolution. However, the loss of the base layer makes the information received from the the enhancement layers useless. The same principle of Layered Coding technique was used in [58] and [61].

A similar concept of LC was used in the work presented in [66] that proposes an image-pixel-position information based resource allocation scheme to transmit wavelet-based compressed images with best effort quality in WMSNs. Using wavelet for image compression will transform the image to coefficients **that** describe its information with different significant-level values that can be further compressed more easily to have at the end some large magnitude coefficients and many small magnitude coefficients with **“0”** bits. These small magnitude coefficients stand for the image-pixel-position information that is more important than the large magnitude coefficients that contains the image-pixel-value information (*i.e.*, brightness). It is shown that the communication loss or bit errors in position information will have significantly higher effect on the overall quality of the received image than the loss or errors in value information. This is because the correct decoding of position data segment depends on the correct decoding of previous position data segments only, but the correct decoding of value data segment depends on previous bit-planes of both position data and value data segments. So, by allocating the scarce resources in WMSN on the position information more than the magnitude value information, position data segments are effectively protected to enhance image quality while value data segments are less protected to improve energy efficiency.

- Predictive Video Coding (PVC) [67], used in MPEG-x and H.26x standards, is based on the idea of reducing the bit rate generated by the source encoder by exploiting data statistics. PVC coding employs two modes for encoding the video: 1) Intra-frame coding mode (I-frame) that is used to reduce the redundancy within one frame by exploiting the spatial correlation in the frame, and 2) Inter-frame coding mode (P-frame) or motion compensated predictive that is used to reduce data redundancy in subsequent frames by exploiting both spatial and Temporal correlation. Performance evaluation of PVC over Stargate and TelosB is conducted in [68] showing the energy consumption in both video compression and transmission.
- Multiple Description Coding (MDC) [69] is used to enhance the error resiliency of video delivery by splitting the multimedia content to two or more independent and equal important streams (multiple descriptions). Each description alone provides acceptable low quality version of the original and combining all descriptions together gives higher resolution. This technique can be used in conjunction with multi path transport approach to achieve load balancing and meet the available bandwidth as shown in [37] and [36].
- Distributed Video Coding (DVC) [70], used for low complexity encoding by shifting the complexity to the sink side, incorporates concepts from source coding with decoder side information for creating an Intra-coded frame along with a side information frame. Therefore, in this technique, multimedia content can be partitioned into multiple streams consisting both intra-coded and decoder side information frames by using simple and low power encoder while the decoder at the destination side can be complex exploiting the availability of the resource such as energy and processing power capability. Two practical DVC encoders are proposed in the literature, Wyner-Ziv (WZ) [71] and PRIZM [72]. DVC coding has been used for WMSN in [73] and [68]. It is shown in [74] through practical implementation of DVC in WMSN that there is a tradeoff between computation and transmission power consumption depending on the encoding schemes used in implementing DVC codec. While a computational intensive scheme, such as discrete cosine transform (DCT), consumes more computational power, it achieves significant compression hence less needed transmission power. On the other hand a less computational intensive scheme, such as a pixel based codec needs less computational power but more transmission power. Therefore, the choice of either scheme (DCT or pixel based) to implement the DVC codec for multimedia content in WMSN depends on the tolerable distortion (quality) and power consumption.

As another methodology for real time video compression and communication over WMSN, Address-Event Representation (AER) is demonstrated in [75]. AER overcomes the limitations of video transmitting over the network in terms of data rate and bandwidth. It is shown in [75] that for streaming video of size 320 × 240 differencing, Crossbow MICAz will be hardly able to deliver the video content at about 2fps at 250 Kbps data rate which it will be hard to understand for the end-user and consume all the communication capabilities of the sensor nodes. Therefore, the proposed algorithm in [75] encodes the video using AE representation, by using custom image sensors capable of detecting intensity-differencing information, and perform a zero-computation compression of the frame-difference video. This compression technique enables sensor nodes to stream temporal frame-difference video over the network at high rates where frame-differencing video can be obtained by subtracting each pixel intensity value in the previous frame from the corresponding pixel intensity value of the current frame. By this way, each pixel value now in the processed video can be presented by only 2 bits (comparing to grayscale video that has a size of 8 bits for each pixel) requiring less bandwidth and also preserving the privacy of the users due to the reduction of intensity information, but it still reveals the motion information. Then frame-difference video can be further compressed by using Address-Event Representation (AER), where events are signaled when changes in pixel intensity reach a certain threshold. In a frame-difference AER image, the pixels that experience large intensity changes will generate events first and more frequently than other events and thus it will be available at receiver side immediately. In order to further compress AER video, it is only required to read less events outputted by the AER algorithm without any computation. It is shown that compression of a 320 × 240-pixel frame-difference images using AER is resulting in 50 times less bandwidth requirement than non-AER scanning video. It is also shown that this technique is only useful with sparse images that have limited number of events (less than 9,000 events) otherwise it will become less efficient than other scanning image techniques.

In [76], eight popular image compression algorithms are reviewed and compared with each other to find the most suitable image compression algorithm for implementation in WMSNs and can meet their requirements including a fast and efficient image processing capability, low memory requirement, high compression quality, less complex system, and low computational load. Image compression process, removing the highly correlated redundant information within the image, contains basically two stages: image transformation stage and entropy coding stage. According to [76], image coding process are categorized into: first generation, which focuses more on how the information contained in the transformed image can be efficiently encoded (such as Discrete cosine transform (DCT), Embedded zerotree wavelet (EZW), Set-partitioning in hierarchical trees (SPIHT), Embedded block coding with optimized truncation EBCOT), and second generation, which put more importance on how the useful information can be extracted and exploited (such as pyramidal coding, Directional decomposition, segmentation based coding, vector quantization). The second generation image compression requires more complex and extensive image processing compared to the first generation image coding as shown in [76]. Among the first generation algorithms, it is shown that SPIHT wavelet-based image compression is the most suitable hardware implemented image compression algorithm for WMSN because it is less complex and needs less computational load and memory allocation.

### 7.2. Collaborative In-network Multimedia Processing

The use of densely deployed sensor nodes in the wireless sensor network provides an inherent protection against normal and provoked system faults. The redundancy of information gathered by neighboring nodes can be exploited for more accurate and robust observation results through effective data fusion and aggregation. In WMSN, the redundancy of information can also be found as overlapping of FoVs of camera sensors located in the same cluster. In addition, the redundancy can be exploited for networking to avoid single-points of communication failure. Therefore, it is necessary to develop efficient and distributed filtering and in-network cooperative processing mechanisms to enable real time retrieval of useful information. For example, in data aggregation, two cameras may collectively fuse their information to obtain a lower bandwidth aggregated result, which is then routed (and possibly fused with other sensor node readings along the way or within the cluster) to the cluster head or the sink.

A distributed filtering architecture is presented in [77] for authoring wide-area sensor-enriched services that supports scalable data collection from high bit-rate multimedia sensors by greatly reducing the bandwidth demands. This architecture (Internet-scale Resource-Intensive Sensor Network, IrisNet) enables the use of application-specific filtering of sensor data near their sources and provides interfaces that simplify collecting and manipulating these data. Also it reduces the processing and bandwidth requirements by detecting repeated computations among the services and eliminating as much of the redundancy as possible. In order to reduce the bandwidth consumed, IrisNet uses distributed filtering in which each service processes its desired sensor feeds on the CPU of the sensor nodes where the data are gathered instead of transferring the raw data across the network. In addition, in order to reduce the computation demands of this approach, IrisNet includes a mechanism for sharing results between sensing services running on the same node.

A collaborative hybrid classifier learning algorithm is proposed in [78] to achieve online vector machine learning for target classification in WMSN. The collaborative hybrid learning algorithm uses progressive distributed computing paradigm for in-network multimedia processing in each cluster, and peer-to-peer paradigm between the cluster heads. The authors show that this algorithm overcomes the disadvantages of using centralized learning paradigm or distributed mobile agent learning paradigm, as consuming too much energy and bandwidth as the case of centralized learning paradigm, and imbalance energy consumption and the need for centralized processing center as the case of distributed mobile agent learning paradigm.

An Artificial Immune System (AIS) based recognition scheme is presented in [79]. The proposed scheme can be used to construct high resolution images from multiple low resolution images captured by multiple sensor nodes in order to improve pattern recognition success rate and image communication energy efficiency. Principal Component Analysis (PCA) is used here for image dimension reduction that extracts only its major or principal components in order to be transmitted to the base station for pattern recognition. In order to reduce the information redundancy and extra number of captured image frames for energy efficient image processing and communication, a sleep/awake schedule algorithm is performed. A node’s sleep/awake status depends on the minimum needed number of image frames to be acquired by the sensor node for successful pattern detection and on the residual energy of the sensor node within a cluster group.

In [80], A compacted probabilistic binary visual classification for human targets in WMSN is presented, where a Gaussian process classifier (GPC) is used for classifier learning instead of support vector machine (SVM). Although SVM has outstanding performance in target classification, SVM causes overhead cost from using parametric optimization algorithm and restricts its application area by getting only the transformed distance between samples and hyper-plane rather than classification probability as in the case of GPC. Also in order to decrease the transmission energy and communication overhead, the GPC classifiers are trained in processing center -single node data processing- and transferred to all sensor nodes rather than using distributed processing. In order to improve the performance of GPC classifier and decrease computing complexity, the raw data -after background subtraction- is refined and the dimension of feature data is compacted by using integer lifting wavelet transform (ILWT) and rough set (RS). And in order to increase robustness and accuracy, committee decision fusion is used to combine individual decisions from different nodes with dynamic weight selection depends on the number of correct classifications of a sensor node and its current energy status.

### 7.3. Traffic Management and Admission Control

The application layer of WMSN also supports, in addition to multimedia processing source coding techniques and in-network multimedia processing, network traffic management and admission control functionalities which are directly related to application-specific QoS requirements. Therefore, based on the traffic class of the application, WMSN needs to provide a differentiated service.

## 8. Cross Layer Optimization

In the previous sections, we discussed the existing proposed solutions designed for WMSNs that follow the classical layered structure of the communication protocol stack. Some of these proposals may achieve a good performance in terms of some metrics related to each of their intended individual layers, but these performance metrics are not jointly optimized to maximize the overall network performance with minimum energy consumption. Moreover, most of these existing solutions do not provide enough support for multimedia applications since the work is done at the lower layers of the communication stack for optimizing functionalities such as optimal routing, reliable delivery, efficient resource management, and other tasks without taking into consideration the especial requirements of handling multimedia content over WMSNs. For example, multimedia compression and source coding algorithms at the application layer should be considered when designing the functionalities at the lower layers and vice versa:

- Cross layer design between multimedia source coding techniques at the application layer and the routing protocol in the routing layer can be exploited for better multipath selection or in-network processing.
- Cross layer design between the routing layer and the MAC layer can allow for packet-level service differentiation or priority-based scheduling and for more power efficient routing mechanisms.
- Cross layer design between MAC layer and the physical layer, especially in the case of using UWB technology. The adoption of the UWB technology as the underlying transmission technique in WMSNs and the potential challenges in this area, appear as an interesting research topic.
- Cross layer design between the routing layer and transport layer especially in the case of multiple paths routing for optimizing the selection of better or most adequate paths that guarantee the required QoS and reliable delivery for each type of multimedia content.

Therefore, in WSNs in general and in WMSNs in particular as they are considered resource-constraint environments, we believe that the correlation characteristics and functionality interdependencies among the layers of the communication stack cannot be neglected and should be exploited for better performance and efficient communication, consequently, cross layer design stands as the most promising alternative to inefficient traditional layered protocol architectures. Recent works in WMSNs show that cross-layer integration and design techniques result in significant improvement in terms of quality of service, energy conservation, and exchanging information between different layers of the communication stack:

A cross layer communication architecture is presented in [24] to provide QoS for WMSN applications based on the Time-Hopping Impulse Radio Ultra-Wide-Band (TH-IR-UWB) transmission technique. This architecture tries to solve the short-comes of using CSMA/CA for the MAC layer in WSN such as the variable and uncontrollable access delays from using random timer, idle listening from using carrier sense, and increased energy consumption occurs due to for example hidden node problem and to provide QoS for WMSN. The proposed system guarantees the end-to-end QoS requirements to handle multimedia content and packet level service differentiation at the network layer in terms of throughput, end-to-end packet error rate and delay by the joint hop-by-hop local decisions of the participating nodes. This is done by an admission control protocol where the sender node that wants to establish connection sends request packet describes its requirements to its neighbors, and among the replies the sender node selects the one who has the most positive advance toward the sink and able to satisfy the needed requirements and this continue iteratively until the end-to-end path is established to the sink. The cross-layer system also provides receiver-centric scheduling based on time hopping sequence of impulse radio of Ultra-Wide-Band MAC and physical layer that allows multiple parallel transmissions, prevents collisions at the receiver node (by using unique TH sequence for each receiver) and saves energy by avoiding idle listening and wasteful transmissions (by turning on exactly on the incoming transmission). This is done by scheduling scheme where each sensor node is responsible to schedule the data packets from its children nodes (closer to sink has higher priority to schedule first) by sending scheduling packets contain different time hopping sequence for each one of them, and also dynamic channel coding to adapt to interference.

Another cross layer design for WMSNs is implemented in [81] where an extension of a routing protocol is presented along with path priority scheduling algorithm for efficient communication of real-time video over WMSNs. The proposed routing protocol is an extension of DGR, Directed Geographical Routing, for constructing multiple disjoint paths in order to enlarge the aggregate bandwidth, facilitate load balancing, and guarantee packet delivery. Using hop-by-hop deviation angle adjustment method, a path can be established using any initial deviation angle specified at the source node, and then other disjoint paths are constructed by changing the value of the deviation angle. To meet the delay constraint of video frames, a path priority scheduling algorithm is used that specifies the number of paths used and assign video sub-streams according to the status of the paths. Using this scheduling algorithm, the weight of each path will be calculated based on the estimated available bandwidth, path delay, and path energy level. Then, by using path weight along with packet priority, shorter delay paths will be used for time-constrained packets while other paths are used for balancing energy and bandwidth usage for other traffic. In case the required bandwidth is larger than the aggregate bandwidth, least priority packets will be dropped. But if the available bandwidth is still not enough, intermediate nodes should decide whether to forward these packets or drop them if they cannot meet their deadline and inform the source node to use either multiple frame selecting or intra-frame refreshing.

In [82], a context-aware cross-layer optimized multi-path multi-priority (MPMP) transmission scheme is proposed in which a multipath routing is used in the routing layer in conjunction with a context-aware multipath selection algorithm in the transport layer. TPGF, Two-Phase geographic Greedy Forwarding, routing that is described in the Routing Section is used to explore maximum number of node-disjoint routing paths while CAMS, Context-Aware Multipath Selection algorithm, is used to choose the maximum number of paths from all found node-disjoint paths for maximizing the delivery of the important data to the sink and guaranteeing end-to-end transmission delay. CAMS algorithm selects the proper routing paths that are suitable for each type of multimedia content based on two types of priority: end-to-end transmission delay based priority for constraint real-time video communication, and context-aware multimedia based priority (image vs. audio) that depends on the importance of multimedia stream which can reflect precisely the event in WMSN.

In [83], a cross layer design is considered for data gathering in WMSN where an adaptive scheme called (RRA) is used to dynamically adjust the “transmission Radius and data generation Rate Adjustment”. Based on the result that the transmission radius of sensor nodes and the data generation rate of sensor nodes are two critical factors in affecting the data gathering performance, the proposed scheme aims to address this research challenge by taking into account the interaction among physical layer, routing layer, and transport layer. RRA scheme first minimizes the end-to-end transmission delay in the network while using minimum data generation rate, and then an optimal transmission radius is calculated in this phase. Then by using this derived transmission radius, the data generation rate can be adjusted to increase the amount of gathered data. Therefore, the cross layer framework of RRA scheme can be summarized into four steps: 1) choosing the optimal transmission radius for sensor nodes at the physical layer, 2) constructing multiple routing paths by using multipath routing protocol like TPGF in the routing layer, 3) selecting the suitable paths from the found paths by the routing protocol at the transport layer, and 4) adjusting the data generation rate of source nodes in the physical layer. Another cross layer design combining network and MAC layers for QoS enhancement in WMSNs is presented in [84]. At network layer, the proposed framework is aiming to find near-optimal paths satisfying the application-specific QoS requirements based on using Multi-Objective Genetic Algorithm (MOGA), while at MAC layer the routing information is used in the MAC algorithm, which is based on CSMA/CA, for QoS-based packet classification and automatic adaptation of the contention window.

## 9. Coverage and Connectivity

Coverage problem becomes critical in WMSN because the deployed multimedia sensors do not have omni-directional (antenna) coverage as the case of the scalar sensors, but they have the feature of capturing direction-sensitive multimedia content with larger sensing radii when there is a line of sight (LOS). Therefore, many proposed algorithms try to exchange local information between neighboring multimedia nodes to determine the most beneficial orientations of their coverage taking into account minimizing the effects of occlusions and overlapping sensing regions and improving the cumulative quality of sensed information from the region of interest. Knowing the overlapping areas between cameras allows exploiting the redundancy in camera sensor coverage in the network and also can be used to track moving objects in the environment.

In [85], a distributed algorithm is proposed to detect the coverage of multimedia nodes and determines their orientation. The algorithm assumes that each multimedia node knows its location information and the location of all the obstacles around it. It starts with broadcasting HELLO messages between the neighbors to exchange the location information of each other and current FoV of each multimedia sensor. By using these information, each multimedia sensor detects the best pose for its field of view using one of the three tests. In Perimeter Test, each multimedia node, with the ability of panning 360° to scan its FoV disk, determines whether a visible FoV (full coverage without occluded or overlapping regions) exists in its FoV disk otherwise the node runs Neighbor-Distance Test. The node can know the overlapping regions by using the received location information from the neighbors. Neighbor-Distance Test examines whether a node has a visible FoV that might be overlapped with other neighbor, but the node needs to find the smallest overlapping FoV by scanning the FoV disk. Finally, if a node could not find a visible FoV even overlapped with other neighbor, the node runs the Obstacle-Distance test to avoid the occlusions from closer obstacles and maximize the visible FoV.

An automated calibration protocol, called Snapshot, is presented in [86]. Snapshot determines and calibrates the location, orientation, and range of camera sensor in WMSNs using the inherent imaging abilities of the cameras themselves and four reference points along with using the principles from optics and geometry in the calibration process. Snapshot uses low-fidelity camera sensors (such as CMUcam or Cyclops) connected with limited computational recourse processors (Crossbow motes or Intel Stargates) and some of the sensors are equipped with a Cricket ultrasound receiver as a wireless calibration device for the reference point. This technique assumes that the intrinsic parameters (focal length, lens distortion, principal point) are known from the manufacture or can be estimated offline prior to deployment, and it only tries to determine the extrinsic parameters (coordinates of the camera, camera orientation, and the FoV of each camera) in order to use as less reference points as possible and reduce the computational cost. In order to determine the location of a camera sensor, the four reference points should be in the visual range of the camera and should not three of them lie along a straight line. By using two reference points and the principles of geometry, the location of the camera lies in closed surface. And by using combination of two reference points each time with the rest of the four points and taking the intersection points of the resulting surface from each calculation with the other, the location of the camera can be estimated after eliminating the false solutions using the optics principles. The orientation of the camera in the three axes (pan, tilt, and rotate) is also determined by using three reference points and the camera location from the previous process. For estimation the visual range of the camera and overlap between other cameras, Snapshot assumes that the size of the internal camera CMOS sensor and the focal length are known offline, and from projection the coordinates of the corners of the CMOS sensor through the camera lens (its location determined from the previous process), the coordinates of a polyhedron or pyramid can be determined using the geometry principles. An object in the volume of the polyhedron is in the visual range of the camera. In order to find the overlapping area between the other cameras, an intersection between any edges of the calculated polyhedron with any other polyhedrons from other cameras is considered as overlapping area. It is shown that this calibration technique gives an error of 1–2.5 degrees in estimating the camera orientation and 5–10 cm error in determining the camera location.

In [87], a scheduling method based on cooperation among nodes for object detection in WMSNs is proposed where the network is clustered based on the overlapping coverage areas of the multimedia sensor nodes. To calculate the overlapping area, the proposed algorithm computes the vertex points of the FoV of each multimedia node by assuming the FoV of a node as an isosceles triangle. A multimedia node’s location represents one of the three vertexes of the FoV and the other two vertexes will be found by the algorithm by knowing the coverage direction angel, orientation angel, and the sensing range of each node. Then the overlapping area is calculated using a decomposition method for intersection polygons. A cluster consists of a subset of multimedia nodes with high overlapping FoV areas where the size of the overlapping area between FoVs of two nodes determines whether they can be in the same cluster. In each cluster, nodes are scheduled to sequentially wake up, capture an image and monitor presence of object, applying object detection procedures such as background subtraction or motion detection and finally go to sleep.

## 10. Security in WMSN

Many applications of WMSN have their additional and special requirements in terms of privacy and security, such as military applications, medical care applications, and other video surveillance systems. In addition to the fact that sensor networks are vulnerable to attacks more easily than the wired networks because of their use of a broadcast medium. Therefore, in order to guarantee message authenticity, integrity, and confidentiality, security in WMSN should be taken into account at the design phase of the network, while at the same time maintaining the efficiency and scalability of the network.

In order to assure authentication and data integrity for multimedia data delivered over WMSN, an energy-aware adaptive wavelet-based watermarking technique for real time image delivery is presented in [88]. This technique embeds additional data called a watermark into some location in an image object so it can be detected later to make an affirmation about the object. The watermarking locations or positions are adaptively chosen by using two thresholds to insert the watermark according to network conditions so that the energy efficiency and security can be achieved. In order to degrade the effect of the distortion of watermarked image, the proposed scheme embeds the watermark into few positions as possible to make it invisible and allocates extra network resources to protect this embedded watermark from high distortion so it can be detectable. In addition, it also embeds watermark coding redundancies into the original image so the watermark becomes more robust to packet loss. The frequency-based (middle band) discrete wavelet transform (DWT) has been selected because it is more robust, easy to recognizable and authenticated at the receiver side, and it reduces computation complexity and process delay by exploiting the correlation of the inter-frames.

In [89], a privacy paradigm called HoLiSTiC is proposed that secures routing and topology information for WMSNs against outsider attacks. The paper assumes a clustered network with some nodes equipped with free-space optical (FSO) capabilities. Also it assumes that the BS and CHs have bidirectional communication links and the camera and transport nodes have unidirectional links. The proposed protocol requires that each node to have an individual key shared with the BS and pairwise keys shared between adjacent visual nodes in a cluster. In addition, every network entity has two pre-deployed network-wide keys. All keys are employed for symmetric cryptography to provide a variety of security services.

A secure data converter architecture is proposed in [90] for WMSN that employs fingerprinting and encryption capabilities for simultaneously digitize and authenticate sensor readings. The proposed architecture suggested hardware modifications to the data converter and aims to reduce the computational complexity of the security algorithms at the aggregation points in the systems that need in-network processing. This can be done by embedding an authenticator payload into the data converter or the modulator output in a way that it is not easily extractable without access to the secret key, and can be used to verify the integrity of the sensor reading.

New trends in security schemes for WMSNs seem to point to energy-aware and lighter-weight security schemes than for traditional networks. Nevertheless, their nodes can use more processor-consuming algorithms than the ones that would be suitable for Scalar Wireless Sensor Networks. This is so, because at least the nodes that have to process multimedia will have more processing capabilities than the typical scalar node.

There have been several papers that propose the use of symmetric cryptography since its lighter processing requirements, but that requires distributed trust by the use of secret sharing or a similar solution, which might be a good solution for some scenarios in scalar WSNs, but not so for WMSNs.

We foresee that for WMSNs asymmetric cryptography will be the chosen approach—over symmetric cryptography—since it makes a lot easier to solve several security problems related to eavesdropping and compromised nodes, and as mentioned before at least some of the nodes will have the required processing power to perform the signature and verification operations.

Watermarking has also been proposed as a way to provide data integrity. Nevertheless, watermarking solutions might be vulnerable to attacks from entities that know how the watermarks are done. In addition, watermarking alone does not solve data authentication. Therefore, we do not foresee watermarking solutions becoming a mainstream approach, but a marginal solution for very specific problems.

Moreover, in order to preserve battery and to save bandwidth, many WMSNs will use some sort of data aggregation. We consider that security and aggregation schemes cannot be devised separately. Therefore, new security schemes will have to be both energy-aware and designed in together with the aggregation scheme.

## 11. Hardware and Testbeds

**Figure 4.**
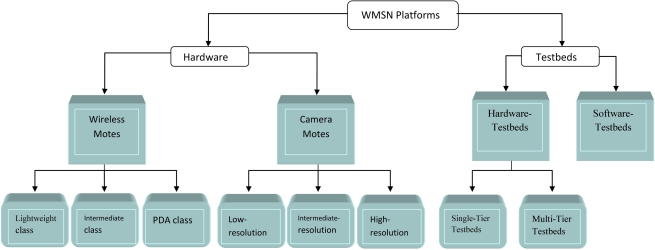
WMSN Platforms Classification.

In order to have the capability of handling multimedia applications in WMSN, the ability to support their requirements and challenges, and to examine and test the proposed protocols and algorithms developed for WMSN, the underlying enabling technology and platforms are required to be more efficient and cover the drawbacks of the existing hardware designed for WSN for detecting scalar events. Therefore, many works have been presented in the literature to modify the existing platform (hardware) or present new hardware implementation and testbeds. These proposed platform and testbeds are more powerful and have more potential to process and handle multimedia traffic efficiently in terms of processing power, memory, data rate, power consumption, and communication capabilities. The work in [91] described the different applications of WMSN and some of the devices and testbeds used in WMSN, but in this section, we introduce most currently off-the-shelf hardware as well as available research prototypes and show their specifications and performance comparing to each other. In addition, we categorize the existing platforms and research prototypes according to their capabilities and functionalities in WMSN as shown in Figure 4 below.

### 11.1. Wireless Motes

**Figure 5.**
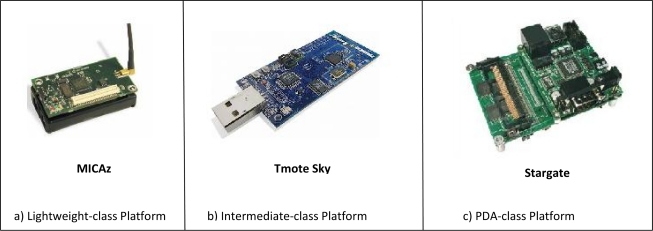
Examples of Wireless Mote Platforms.

There are several devices of wireless motes that can be used as WMSN motes and most of them are available in commercial products as shown in Figure 5. Depending on their processing power and storage capacity, these wireless motes can be classified into three groups:

1. **Lightweight-class Platforms:** The devices in this category are designed initially for detecting scalar data, such as temperature, light, humidity *etc.*, and their main concern is to consume less amount of energy as possible. Therefore, these devices have low processing power capability and small storage and most of them are equipped with a basic communication chipset (e.g., IEEE 802.15.4 on CC2420 radio). The CC2420 chipset only consumes 17.4 and 19.7 mA for sending and receiving respectively and has maximum transmit power of 0 dBm with data rate of 250 Kbps. Table 4 shows examples of lightweight-class wireless motes, Mica-family motes [92] and FireFly [93], and compares their specifications.
2. **Intermediate-class Platforms:** The devices in this group have better computational and processing capabilities and larger storage memory than lightweight-class devices. However, they are also equipped with low bandwidth and data rate communication module (e.g., CC2420 chipset which is IEEE 802.15.4 compatible). Tmote Sky [94] is an example of Intermediate-class mote designed by Moteiv (Sentilla) that uses low power 8 MHz 16-bit MSP430 F1611 RISC processor from Texas Instruments featuring 10kB of RAM, and 48kB of flash. Tmote Sky uses Chipcon CC2420 radio for IEEE 802.15.4/ Zigbee for maximum data rate of 250 Kbps. Tmote Sky has been used to implement camera mote with CITRIC [95] and CMUCam3 [96].
3. **PDA-class Platforms:** The devices in this category are more powerful in terms of computational and processing power and they are designed to process multimedia content in a fast and efficient manner. These devices can run different operating systems (e.g., Linux, TinyOs, and run Java applications and .NET micro frameworks) and support multiple radios with different data rates (e.g., IEEE 802.15.4, IEEE 802.11, and Bluetooth). However, these devices consume relatively more energy. Stargate and Imote2 are examples of PDA-class platforms. Stargate board [97], designed by Intel and manufactured by Crossbow, uses 400 MHz 32-bit Marvell’s PXA255 XScale RISC processor with 32 MB of Flash memory and 64 MB of SDRAM and runs Linux operating system. It can be interfaced with Crossbow’s MICA2 or MICAz motes for IEEE 802.15.4 wireless communication as well as PCMCIA IEEE 802.11 wireless cards or compact Flash Bluetooth. Thus, Stargate board can be used as a sensor network gateway, robotics controller card, or distributed computing platform. It forms a camera mote when it is connected with camera device (e.g., webcam) as shown in [98–100]. Imote2 [101], also designed by Intel and manufactured by Crossbow, is a wireless sensor node platform built around the low-power 32-bit PXA271 XScale processor and integrates an 802.15.4 radio (CC2420) with a built-in 2.4 GHz antenna. It can operate in the range 13–416 MHz with dynamic voltage scaling and includes 256 KB SRAM, 32 MB Flash memory, 32 MB SDRAM, and several I/O options. It can run different operating systems such as TinyOs and Linux with Java applications and it is also available with .NET micro framework. It integrates many I/O options making it extremely flexible in supporting different sensors including cameras, A/Ds, radios, *etc.* The PXA271 processor includes a wireless MMX coprocessor to accelerate multimedia operations and add media processor instructions to support alignment and video operations. Imote2 has been used as a camera mote in [102, 103].

### 11.2. Camera Motes

In order to reduce the amount of resources required by transmitting multimedia traffic (images, videos) over WMSN, the multimedia content should be intelligently manipulated and processed using appropriate compression and coding algorithms along with other application-specific multimedia processing such as background subtraction, feature extraction, *etc.* However, most of these algorithms are complex and require high computational and processing power as well as larger memory for buffering frames. Sometimes, these requirements cannot be satisfied with the only resources offered by the wireless motes, which we mentioned before, especially if they require floating-point operations for efficient multimedia processing. Therefore, camera sensor may coupled with additional processor (microcontrollers, DSPs, FPGAs, *etc.*) and memory resources before relaying the processed data to the wireless mote for wireless communication. Nevertheless, the additional processor and memory resources require more energy consumption and cost and this makes a tradeoff between energy consumption and cost on one side with computational power and traffic amount on the other side. It has been shown in [104] that the time needed to perform relatively complex operations on a 4 MHz 8-bit processor such as the ATmega128 is 16 times higher than the time needed with a 48 MHz 32-bit ARM7 device, while the power consumption of the 32-bit processor is only six times higher. Hence, this indicates that the powerful processor (such as 32-bit ARM7 architecture) is more power-efficient in multimedia applications. Table 5 shows the existing multimedia platforms and research prototypes for WMSN and compares between their specifications.

**Figure 6.**
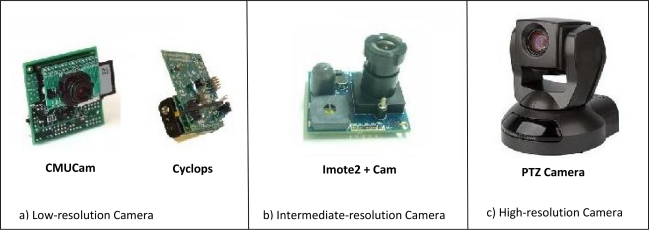
Examples of Camera Mote Platforms.

¿From Table 5, we can conclude that camera motes have different capabilities (resolution, processing power, storage, and others) and accordingly, depending on their capabilities and features, they have different functionalities and play different rules in the network. For example, low resolution cameras can be used at the lower-tier of multi-tier network for simple object detection task to exploit their low-power consumption feature that allows them to be turned on most of the time (or in duty cycle manner). Cyclops, CMUCam3, and eCam [105] are examples of *low-resolution* cameras. Intermediate and high resolution cameras can be used at higher-tiers of the network for more complex and power-consuming tasks, such as object recognition and tracking. These types of cameras consume more power and hence there are only woken up on-demand by lower-tier devices, e.g., in case detecting an object of interest. Webcams, attached for example with Stargate board or Imote2, can be considered as *intermediate-resolution* cameras, while PTZ cameras used in [98] is an example of *high-resolution* camera. Figure 6 shows commercial product examples of camera mote platforms used in WMSNs.

Cyclops [106] is a small camera device developed for WMSN. Cyclops is compatible with the computationally constrained wireless sensor nodes (motes) and exploits the characteristics of CMOS camera sensors as they are low power, low cost, and small size sensors. Cyclops platform isolates the requirement of camera module for high speed data transfer from the low-speed capability of the embedded controller and provides still images at low rates. It is designed to be interfaced with the common motes used in wireless sensor networks such as MICA2 and MICAz. Cyclops hardware architecture consists of an imager (Agilent compact CIF CMOS ADCM-1700), an 8-bit RISC ATMEL ATmega128L micro-controller (MCU), a Xilinx XC2C256 CoolRunner complex programmable logic device (CPLD), an external 64KB SPRAM, and an external 512KB Flash. The MCU controls Cyclops to capture images and communicate with host to provide image interface, while CPLD provides high speed clock, synchronization, and memory control required by the image capturing that cannot be satisfied by using a lightweight processor. So, CPLD acts as a lightweight frame grabber to provide on-demand access to high speed clocking at capture time and perform a limited amount of image processing such as background subtraction or frame differentiation. Cyclops firmware is written in nesC language and runs under TinyOS operating system. In addition to the libraries provided by TinyOS, Cyclops also provides primitive structural libraries (such as matrix operation libraries or histogram libraries) and advanced or high-level algorithms libraries (such as coordinate conversion and background subtraction). The authors show in the performance analysis that Cyclops is a low power device and its energy consumption depends on the power consumption of different states (such as image capturing, memory access, micro-controller processing, sleep *etc.*) and their time duration as well as on the input image size and the ambient light intensity.

FireFly Mosaic, a vision-enabled wireless sensor platform and image processing framework presented in [107], uses camera motes consisting of FireFly wireless node coupled with a CMUcam3 camera sensor. The FireFly nodes run the Nano-RK real-time operating system and communicate wirelessly using the RT-link collision-free TDMA-based protocol. FireFly Mosaic is designed to be low-cost, energy efficient, and scalable compared to the centralized wireless webcam-based solution. The used RT-link TDMA-based link wireless communication provides tight global time synchronization to prevent collisions and save energy while Nano-RK operating system provides hooks for globally synchronized task processing and camera frame capturing. While the network communication relies on TDMA-based link layer, the internal communication between the camera and the wireless node is based on the Serial Line IP (SLIP). The CMUcam3 camera of FireFly Mosaic consists of CMOS (OmniVision OV6620) camera ship capable of capturing fifty 352 × 288 color images per second, frame buffer (Averlogic AL440b FIFO), and 23-bit (LPC2106 ARM7TDMI) microcontroller running at 60MHz with built-in 64KB RAM and 128KB Flash memory. Also CMUcam3 has four on-chip servo controller outputs which can be used to actuate a pan-tilt device. In the other hand, FireFly sensor node has a low-power ATMEL ATmega 1281 8-bit processor with 8KB RAM and 128 KB Flash memory, connected with Chipcon CC2420 802.15.4 radio capable of transmitting a 250 Kbps for up to 100 meters. CMUcam3 is an open-source camera comes with several libraries (named CC3) and example applications such as JPEG compression, frame differencing, color tracking, convolutions, edge detection, connected components analysis, and a face detector. This several image processing algorithms can be run at the source and only the results may be sent over the multi-hop wireless channel to the FireFly gateway. CMUcam3 can be also interfaced with other type of sensor nodes such as TolesB and Tmote Sky motes running different operating systems.

Wica [108] is another camera mote designed for wireless multimedia sensor network. The wireless camera mote is based on an SIMD (Single Instruction Multiple Data) video-analysis processor and an 8051 micro-controller as a local host, and it is using the IEEE 802.15.4 standard (ZigBee) for its wireless communication. The camera consists basically of four components: one or two VGA color image sensors, an SIMD processor for low-level image processing, a general purpose processor for intermediate and high-level processing, and control and communication module. The SIMD processor is of type IC3D from Philips’Xetal and it consists of Linear Processor Array (LPA) with 320 RISC processors. 8051 controller from ATMEL is used as a general purpose processor and it includes 1.79MB RAM, 64KB Flash, and 2KB EEPROM to store the parameters and instruction code for IC3D processor. Both processors are coupled using a 128 KB dual port RAM that enables them to work in a shared workspace asynchronously. The Aquis Grain Zigbee from ChipCon’CC2420 transceiver implements the wireless communication module. The multimedia processing in this camera sensor mote is divided into three levels: low, intermediate, and high-level image processing. Low-level image processing (pixel level) is manipulated by the SIMD processor and it is associated with typical kernel operations such as convolutions, data dependent operations using neighboring pixels, and initial pixel classification. The intermediate and high-level image processing (object level) are done by the general purpose processor because it has the flexibility to implement complex software tasks, run an operating system, and do networking application.

In [102] the authors present a camera mote for behavior recognition in wireless multimedia sensor networks based on biologically inspired address-event imagers and sensory grammars. In Address Event Representation (AER), the camera networks operate on symbolic information rather than images by filtering out all redundant information at the sensor level and outputting only selected handful of features in address-event representation. This leads to minimize power consumption and bandwidth (they only consume a few *μ*W of power in active state and use different computation model that is faster and more lightweight than conventional image processing techniques), and helps to offer privacy concerns as certain features are being transmitted. Then the output of the AER imagers can be connected into the sensing grammar that converts low-level sensor measurements to higher-level behavior interpretation based on probabilistic context free grammars (PCFGs). PCFGs are very similar to the Hidden Markov Models and they are used because of their expressiveness, generative power, and modularity. The authors developed three different platforms to experiment the above techniques where each platform is built on top of the XYZ sensor node [109]. XYZ uses an OKI ML67Q5002 processor based on ARM7TDMI core running at 58 MHz. The processor has 32 KB of internal RAM and 256 KB of Flash, and there is additional 2Mbit memory available on-board. The first platform is (XYZ-ALOHA) an XYZ sensor node with ALOHA image sensor that is composed of four quadrants of 32 × 32 pixels and it is able to generate 10,000 events in 1.3sec with a power consumption of 6 *μ*W per quadrant. The ALOHA image sensor uses the simple ALOHA medium access technique to transmit individual events to a receiver. The second platform is (XYZ-OV) an XYZ sensor node with a camera sensor from Omnivision that can capture images at resolution of VGA (640 × 480) and QVGA (320x240). Currently, imote2 has been used with Omnivision OV7649 camera as a third platform. The paper shows an example for assisted living application where the prototype network (imote2-OV) was able to distinguish between “cooking” from “cleaning” actions done by a person in a kitchen.

An energy-efficient smart camera mote, called MeshEye [110], is proposed for distributed intelligent surveillance application in WMSN. MeshEye mote architecture is designed to support in-node image processing, with sufficient processing power capabilities, for distributed intelligent algorithms in wireless sensor network of two tiers while minimizing component count and power consumption. In the first tier, a low-resolution stereo vision system is used to determine position, range, and size of moving objects in its field of view. The second tier contains high resolution cameras that are triggered in case of detecting objects by the first tier. The MeshEye mote has an Atmel AT91SAM7S microcontroller board with 64 KB SRAM and 256 KB Flash memory, and the mote can host up to eight kilopixel imagers (Agilent ADNS-3060) and one VGA camera module (Agilent ADCM-2700). The wireless communication module uses CC2420 2.4GHz IEEE 802.15.4 RF transceiver that can support up to 250 Kbit/s. Although the supported data rate is not high enough for multimedia streaming, the authors show that it is still possible by conducting in-node intermediate-level visual processing for efficient image compression and/or descriptive representations (such as axis projection, color histogram, or object shape). Also the authors present a basic power model that estimates the energy consumed in different operation modes by the battery-powered MeshEye mote.

In [100] the design, implementation, and performance of video-based sensor networking architecture using visual sensor platform (called Panoptes) are introduced for delivering high quality video over 802.11 wireless networks. The initial developed hardware platform of Panoptes was the Applied Data Bitsy board utilizing the Intel StrongARM 206-MHz embedded processor connected with Logitech 3000 video camera via USB. Because of the limitations found by using this design such as slow video capturing, low processing power, high power consumption, and small available memory, the authors prefer to use Crossbow Stargate platform that has twice processing power more than the Bitsy board, consumes less power, and has smaller size. The second design of Panoptes node based on Stargate platform offers video capturing at reasonable frame rate (more than 15 fps) using Logitech 3,000 pro webcam. After video capturing, the software module in Panoptes provides video frame compression, both spatially and temporally, using JPEG, differential JPEG, and conditional replenishment. Also the software module in Panoptes provides other functionalities such filtering for dropping similar video frames, buffering management, and adaptation for network status. These functionalities can be accessed simply by function calls based on Python language by which the user can chose the preferred algorithm or method in each subcomponent (e.g., selecting compression algorithm or filtering method) at the run time without the need of manually reprogramming the nodes. At the end, the authors show the implementation and performance of a video aggregation application and some algorithms (such as prioritizing buffer management algorithm and bit-mapping algorithm for video querying) using Panoptes nodes.

As an example of medium-resolution camera mote, an embedded camera mote platform [14] based on Fox board has been introduced for wireless multimedia sensor network applications. The designed platform has several sensors including GPS positioning receiver, current consumption sensor, and image sensor beside the wireless transceiver. The Fox board LX416 has 100 MHz CPU, 4MB Flash, and 16MB of RAM running GNU/Linux as operating system and because of these capabilities it is attended to be use for high-level device of multi-tier model. The platform can be connected via USB ports with webcam (QuickCam Zoom or Labtec Webcam) and Bluetooth dongle. The designed platform is using Bluetooth (IEEE 802.15) for data transmission, rather than 802.11 comparing to other high-level platforms like Panoptes or the one used in SensEye, because of the availability of USB-Bluetooth dongles, open-source software support and moderate power consumption. The current consumption sensor is used as an energy analyzer to study energy consumption of nodes during image transmission and it is shown in the experimental results that image grabbing and transmission needs more power than image routing.

The work in [15] proposes the design of a wireless video sensor node, called MicrelEye, for video processing and image classification in wireless multimedia sensor networks. The device is equipped with a VGA CMOS (OV7640 from Omnivision) image sensor, a reconfigurable processing engine, and a Blue tooth 100m transceiver. The design is intended to be low-cost low-power multimedia sensor node that can support dynamic reconfiguration capabilities and local processing for multimedia content, such as back ground subtraction, image recognition and classification, before wireless transmission. An optimized hardware-oriented support vector machine-like (SVM-like) algorithm called ERSVM is used for image classification process. The devise uses a System on Chip (SoC) for the processing engine, ATMEL FPSLIC, which includes AVR 8-bit RISC MCU, 40K gates FPGA, and 36KB SRAM. An external 1MB SRAM is also added to provide the required memory resources for multimedia processing and enable parallelized computation between hardware and software. With these specifications, the device targets a power budget of 500 mW and supports people detection at 15 fps at QVGA (320 × 240) image resolution. For wireless communication, a 100 m LMX9820A Bluetooth transceiver has been used because of its low power of consumption, the ease to interface MicrelEye with other devices, and its high data rates (up to 704 kbps). In Video processing algorithm, the FPGA starts the process by acquiring frames from the image sensor, and then a back ground subtraction is done on each acquired frame. After that the region of interest (ROI), 128 × 64 subimage, is extracted and stored into on-chip memory to be processed by MCU. The MCU will conduct on the ROI a feature extraction to form the feature vector and image classification using ERSVM algorithm.

A camera mote called CITRIC is developed in [95] for wireless multimedia sensor networks to enable in-network processing of images in order to reduce communication overheads. The hardware design of the camera platform consists of camera sensor, PDA class processor, 64MB RAM, 16MB Flash, and microphone. The camera sensor is a 1.3 megapixel OmniVision OV9655 camera that can support different image resolution -from SXGA (1280 × 1024) through VGA, CIF to 40 × 30-outputting 8bit/10bit images at a rate of 30 fps in VGA and lower resolution and typically consumes 90mW in active state. The processor is PXA270 frequency-scalable (up to 624MHz) fixed-point, and it has 256KB internal SRAM and a wireless MMX coprocessor to accelerate multimedia operations. Then this camera device is connected to standard sensor network mote (Tmote Sky) to form the wireless camera mote, CITRIC, which communicates over the IEEE 802.15.4 protocol at a rate of 250 Kbps. The camera mote first performs pre-processing functions on the captured images from the camera sensor and then sends the results over the network to a central server. Also the paper proposes a back-end client/server architecture to provide user interface to the system and support further centralized image processing. The authors implement three applications over the proposed platform which are image compression, target tracking, and camera localization. In image compression application, it is shown that Compressed Sensing (CS) using random matrices provides unique advantages in lossy compression than JPEG standard when both are implemented using the integer DCT implementation that is supported by the fixed point arithmetic processor. The single target tracking application is implemented via background subtraction using frame differencing. Then the foreground pixels are processed for identification and tracking. An implementation of Dual-Camera sensor is presented in [103]. Each of them comprises of a low-power and high-power tier and they are physically connected together to have similar FoV. The low-power camera sensor node (Tier-1) consists of a MICAz mote equipped with a low fidelity Cyclops camera sensor, and a 1GB NAND flash for storing images. The high-power camera sensor node (Tier-2) consists of a more-powerful platform, imote2 equipped with a high fidelity Enalab camera (OV7649 CMOS camera supports color VGA (640 × 480) resolution), and a 1GB SD card for image storage. The system uses the low-power Tier-1 for object detection and the high-power Tier-2 for energy efficient object recognition and classification. The wireless communication is based on IEEE 802.15.4 Zigbee standard at 2.4 GHz.

### 11.3. Testbeds

To evaluate different protocols and algorithms pertaining for different networking layers (transport, network, MAC, or physical layer) of wireless multimedia sensor network or test various applications over the WMSN, researchers may perform analytical analysis, conduct experiments, or use simulations. Sometimes, analytical analysis neither gives an accurate model for such complicated wireless system nor truly depicts the behavior of real-time wireless networks. Also, in many cases, tests and experiments in wireless sensor network in general and in wireless multimedia sensor network in particular are somehow complex and time-consuming, and hard to be re-conducted by other researchers. For these reasons, simulation has been the preferred methodology for many researchers in the wireless multimedia sensor network domain. However, the existing simulators have many defects and are unable to model many critical characteristics of real-time wireless systems. Also, because of not following the scientific research standards in conducting of such simulation studies, simulation results are sometimes doubtful and have less credibility [111]. For these reasons and in order to minimize the differences in results between theoretical and practical approaches, which will significantly affect the behavior of real-time systems, testbeds have been increasingly used by the researchers and developers to evaluate their proposed algorithms and applications.

WMSN testbeds are used for better understanding and satisfying the practical and technical challenges of networks deployed in real-time systems. While testbeds have become the preferred method for testing and evaluating with wireless multimedia sensor network applications, they also provide means for integrating several individual sensors on a common wireless platform in a controlled and instrumented environment. Thus, research on experimental testbeds with current hardware and software platforms, allows users not only to demonstrate applicability and evaluate application-level and network-level performance metrics (e.g., detection probability, end-to-end delay, jitter, quality of received multimedia streams, *etc.*) in real environments, but also to validate research prototypes. Compared with conducting real-time experiments and field deployments, testbeds give considerable efficiency in testing potentially long-time experiments, which is important in debugging, validation, and integration phases of reliable wireless multimedia sensor networks. WMSN testbeds can be classified into two categories, *Software Testbeds* and *Hardware Testbeds*. Table 6 illustrates the existing software and hardware testbeds found in the literature and summarizes their specifications and important features.

#### Software Testbeds

To facilitate advanced research in wireless multimedia sensor network technology, software driver interfaces and libraries are designed to help researchers in testing and evaluating various algorithms and applications through using easy-to-use Application Program Interfaces (APIs) and functions. These APIs and functions provide testing environment through abstraction layers that hide the low-level details of the underlying hardware in order to enable easy and fast development of multimedia sensor network applications.

WiSNAP [112] is a Matlab-based software testbed designed for wireless multimedia sensor networks, where the developers can test and evaluate algorithms and applications using its standardized and easy-to-use Application Program Interfaces (APIs). WiSNAP provides a Matlab framework as a high-level and powerful programming environment for implementing interfaces to the existing wireless motes and image sensors though simple easy-to-use functions and libraries. These functions and libraries hide the internal details of dealing with mote or sensor specific interfaces from the end-users and provide the users with many powerful and rich image processing tools. Currently, WiSNAP includes device libraries for Agilent’s ADCM-1670 camera module, Agilent’s ADNS-3060 optical mouse sensor, and Chipcon’s CC2420DB IEEE 802.15.4, but it can be extended to include and support any kind of sensor or wireless mote as it is an open source architecture. WiSNAP consists of two application program interfaces: 1) an image sensor API that enables frame capturing from image sensors after identifying the type of image sensor and number of frames, and 2) a wireless mote API that provides access to wireless motes through functions for initialization and MAC packet transmission and reception. Then these set of functions provided by the mentioned APIs are matched with the corresponding device libraries that lie below the API layer in WiSNAP program stack and provide a set of hardware-dependent functions (such as Agilent ADCM-1670 image sensor). Two application examples of using WiSNAP development platform are presented for event detection and node localization. Event detection is based on tracking of the number of changed pixels that exceed a certain threshold between successive image frames. For node localization, the distance is estimated using the received signal strength indicator RSSI from the node and the direction is calculated by extracting the relative angle of a continuously blinking LED on that node from the captured images using frame differencing of adjacent frames.

Also in [102], a software testbed based on address event representation of image sensing is developed. The software testbed consists of an emulator of AER imagers - written in VisualC++ and runs under windows. the AER Emulator takes an 8-bit grayscale input stream from a COTS USB camera and outputs a queue of events to a text file. The AER classification is done in a way similar to the Hidden Markov Models (HMMs). At the receiver side, the image array can be obtained by converting the event frequency data into the original feature (e.g., light intensity) using two ways, Histogram reconstruction or Inter-event reconstruction.

#### Hardware Testbeds

Hardware testbeds involve deploying of hardware devices, such as multiple types of cameras with different resolutions and image-processing abilities, and wireless communication hardware that may support multiple standards and different data rates. Besides that, hardware testbeds provide supporting software for data monitoring and user interface. Depending on the hierarchal organization supported by the network, Hardware testbeds can be further divided into single-tier or multi-tier testbeds:

**A) Single-Tier Hardware Testbeds:** Meerkats [99] is a testbed of wireless network of battery-operated camera nodes used for monitoring and surveillance of wide areas. The Meerkats node, which is based on Stargate board and using 802.11b wireless card, is equipped with sufficient processing and storage capabilities (when compared to a Cyclops node) for running relatively complex image processing algorithms. The goal of the work is to measure the tradeoff between application-specific performance and power efficiency (or network life time) for a given resource management strategy. Meerkats currently composed of eight visual sensor nodes, each of which consists of -as we mentioned-a battery powered Crossbow Stargate board, which has an XScale PXA255 CPU (400 MHz) with 32MB flash memory and 64MB SDRAM, connected with a Logitech QuickCam Pro 4000 webcam via USB and IEEE 802.11b PCMCIA wireless card. The Stargate platform is selected for the Meerkats node because it is running an open source operating system (Linux kernel 2.4.19), it can be easily connected to a webcam, the image sensor in Meerkats node, and it provides sufficient processing and storage capabilities. A laptop acts as base station or information sink running a multithreaded server program. For energy conservation, the Meerkats node operates according to a specific duty cycle in which it switches periodically its components (processor, camera, radio) into different operation states (sleep, idle, active) and performs a specified tasks. Meerkats’s energy performance evaluation can be seen at [113]. The Meerkats node performs all the image-related tasks such as image acquisition, processing, and compression when it is in the active state. For example, for event detection, the moving blobs in the image are detected using a fast motion analysis algorithm and the relevant information is compressed using JPEG standards. The communication, based on multi-hop routing, between Meerkats nodes are established using the Dynamic Source Routing (DSR) routing protocol through IEEE 802.11b links.

The Mobile Emulab network testbed [114] provides a remotely accessible mobile wireless and sensor testbed. The mobile testbed can provide accurate positioning and monitoring using video camera equipments, and enable automated experiments by both on-site and off-site users by using open-source software and COTS equipments. The testbed consists of Acroname Gracia robots attached with Mica2 motes, Stargate boards with IEEE 802.11b cards, and low-cost Hitachi KP-D20A cameras. The testbed is used in an indoor field of sensor-equipped motes and webcams, and can provide simple path planning as well as vision-based tracking system accurate to 1 cm. Mobile Emulab testbed allows remote user to position the robots, control all the computers and network interfaces, run arbitrary programs, and log data in a database. Emulab testbed allow, through precise positioning and automation, quick evaluation of localization and mobility protocols in sensor-driven applications.

Low-cost, vision-enabled, and flexible autonomous mobile robots were designed in the Explorebots testbed in [115] for indoor experimentation on multi-hop ad hoc and sensor networking. The wireless robots are equipped with MICA2 sensor motes for sensing and wireless communication, in addition to built-in electronic compass, velocity and distance sensors, motor movement control, and sonic-based ranging sensors that can be used for navigation. The hardware components of the robot consist of mobile platform, 8-bit Rabbit semiconductor R3000 programmable microprocessor with Flash memory, a 320 × 240 pixel X10 Cam2 color camera, sensing elements, communication devices, and batteries. Explorebots testbed has been used for target localization experiments by processing the sound and light sensors outputs to guide the robots towards the target source, in addition to validate hybrid routing protocols.

**B) Multi-Tier Hardware Testbeds:**

IrisNet [116], internet-scale resource-intensive sensor network services, is an example of wireless multimedia sensor network Multi-Tier Hardware testbed that provides shared internet-scale long-lived software platform for many sensor applications. IrisNet has been designed to overcome the difficulties of building large-scale distributed networks comprise of many scalar and visual sensors, and the challenges of dealing with large volumes of collected data. Therefore, the proposed platform enables the creation of a planetary infrastructure of multimedia sensors and enables application-specific processing of the collected data by these sensors using their processing capabilities. IrisNet allows user to query the collected information, stored in distributed XML database infrastructure close to its sources, by using internet-like queries. IrisNet also provides a number of multimedia processing primitives that new applications can use as building blocks such as camera calibration, key-points or reference points implementation, and image stitching. The architecture of IrisNet is two-tiered: Sensing Agents tier (SAs) for data collection and filtering, and Organizing Agents tier (OAs) for data storage and querying. There are three steps in order to develop an application using IrisNet. First, the application developer creates the sensor database XML schema that defines the attributes, tags, and hierarchies used to describe and organize distilled sensor data. Second, the application developer writes the software running in the SAs (called senselet) to filter the collected sensory data and update the database defined by the schema. Third, the application developer provides an application-specific front end interface for end users to access the application.

In [98], the design and implementation of SenseEye is presented, a multi-tier network of heterogeneous wireless sensor nodes and cameras. SenseEye is designed for surveillance application in WMSN where resource-constrained low-power elements are employed to perform simpler application tasks while more capable high-power elements are used for more complex tasks. The work aims to exploits the advantages of multi-tier sensor network comparing to a single-tier network such as low cost, wide coverage, high functionality, and high reliability by proposing numerous mechanisms and optimizations for object detection, object localization, inter-tier wakeup, object recognition, and tracking. SenseEye is implemented in three-tier network consisting of four types of camera sensors where nodes within each tier are assumed to be homogeneous while different tiers are assumed to be heterogeneous with respect to their capabilities. The processing power, networking capabilities, and imaging resolution improve from the lower tier to the higher tier at the expense of increased power consumption. The lowest tier consists of low-power sensor motes such as MICA2 equipped with low fidelity and resolution camera sensors such as Cyclops or CMUcam3. The second tier consists of Stargate nodes equipped with higher fidelity and medium resolution webcams. The third tier contains a sparse deployment of high resolution pan-tilt-zoom (Sony SNC-RZ30N) cameras connected to embedded PCs. In this system, no base station is assumed and the communication between Tier 1 and Tier 2 is low rate through 900 MHz radio while the communication between Tier 2 and Tier 3 is done through 802.11 radio. The main design principles of the proposed system are mapping each task to the lowest powerful tier that has the sufficient resources to accomplish the needed tasks reliably within the required latency, exploiting the wake-up on-demand and triggering of the higher tier nodes only when necessary in order to save energy, and exploiting the information redundancy from overlaps in cameras coverage to improve energy-efficiency and performance (e.g., overlaps camera coverage information can be used in object localization for intelligently wake up the correct nodes in the higher tier). The paper shows a practical example of the proposed system where the nodes in the first tier are always turned on or duty-cycled (woke up periodically) and used for object detection (through simple frame differencing) and then, in case of detecting an object, the nodes try to localize the detected object exploiting the information redundancy from overlapping camera coverage and using triangulation techniques for localization. After that, the nodes in Tier 1 woke up the nodes in Tier 2, which are close the detected object (within their FoV), that in turn perform object recognition by capturing photos of the object, identifying object features, and searching the database for a match. Finally, the corresponding nodes in the Tier 3 are woken up to perform object tracking with the help with the other tiers as the detected object is moving.

The WMSN-testbed [17] at the Broadband Wireless Networking (BWN) Laboratory at Georgia Tech is based on commercial off-the-shelf (COTS) advanced devices and has been built to demonstrate the efficiency of algorithms and protocols for multimedia communications through wireless sensor networks. The testbed is integrated with the scalar sensor network testbed, which is composed of a heterogeneous collection of Imote2 and Micaz motes from Crossbow. The testbed allows the integration of heterogeneous devices in experimental testbeds and includes three different types of multimedia sensors: low-end imaging sensors, medium-quality webcam-based multimedia sensors attached with Stargate boards, and pan-tilt cameras mounted on Acroname GARCIA mobile robots. The testbed uses both IEEE 802.15.4 and IEEE 802.11b for wireless communication and it is capable to deliver JPEG video streaming in QCIF format (176 × 144) at 15 fps.

## 12. Conclusions

In this paper, we discussed and surveyed in detail the research carried on Wireless Multimedia Sensor Networks (WMSNs). We analyzed the major technical challenges and research issues in designing algorithms, protocols, architectures, and hardware for WMSN. We discussed most of the existing solutions for WMSN at the different layers of the communication stack: physical, MAC, routing, transport, and application along with the possible cross layer implementation. Furthermore, we discussed other complementary research issues in WMSN such as coverage and security issues. Finally, we surveyed and classified the existing off-the-shelf devices, prototypes, and testbeds implemented for WMSNs.

## Figures and Tables

**Table 1.** Specifications of the Physical Layer Standards in WMSNs.

	**ZigBee**	**Bluetooth**	**802.11**	**UWB**
**Data Rate (max)**	250 Kbps	1 Mbps (vl.2) 3 Mbps (v2.0)	54 Mbps	250 Mbps (up to now)
**Output Power**	1 – 2 mW	1 – 100 mW	40 – 200 mW	1 mW
**Range**	10–100 meters	1 – 100 meters	30 – 100 meters	< 10 meters
**Frequency**	2.4 GHz or 915 MHz or 868 MHz	2.4 GHz	2.4 GHz	3.1 GHz - 10.6 GHz
**Code Efficiency**	76.52%	94.41%	97.18%	97.94%
**No. Nodes**	< 65000	7	30	-

**Table 2.** A Comparison between MAC Layer Protocols. Grey rows indicate that the MAC protocol is designed for WSNs but not specifically for WMSNs.

MAC Protocol	(Single/Multi)-Channel	Contention-(based/free)	Diff. Service	Topology	Cross-Layering
T-MAC [21]	single	free (scheduling-based)	no	clustered	no
S-MAC [20] B-MAC [25] MMAC [26]	single single multi	free (scheduling-based) based based (IEEE 802.11)	no yes no	clustered flat flat	no yes no
MMSN [27]	multi	based	no	flat	no
COM-MAC [19]	multi	free (scheduling-based)	yes	clustered	-
Diff. Service Model [28]	-	-	yes	flat	-
MAC Protocol in [22]	single	based (CSMA)	yes	flat	no
Cross-layer Architecture in [24]	multi (UWB)	free	yes	flat	yes
EQ-MAC [29]	single	based (collision-free)	yes	flat (static)	yes
Node Admission [30]	single	free (TDMA)	no	flat	yes
UWB Technology in [16]	multi	free	yes	flat	yes

**Table 3.** Methodologies used in proposed routing protocols for WMSN.

**Routing Protocol**	**Methodologies**				
	**Multi-path**	**Multi-channel**	**Geographic**	**Multi-radio**	**Hierarchical**
ASAR [32]	✓				✓
TPGF [33]	✓		✓		
Swarm-based LANMAR [35]				✓	✓
Radio-Disjoint routing [36]	✓				
Modified Direct Diffusion [37]	✓				
PPDD-based QoS routing [38]	✓	✓			
M-IAR [40]			✓		
Multimedia-aware MMSPEED [42]	✓		✓		
QuESt [45]	✓				

**Table 4.** Comparison of the Features of Wireless Motes.

	Wireless Mote	Microcontroller	Memory		Radio	Data Rate
			RAM	Flash Memory		
Lightweight-class	Mica2	ATmega128L (8 bit) 7.37 MHz	4 KB	512 KB	CC1000	38.4 Kbps
	Mica2Dot	ATmega128L (8 bit) 4 MHz	4 KB	512 KB	CC1000	38.4 Kbps
	MicaZ	ATmega128L (8 bit) 7.37 MHz	4 KB	512 KB	CC2420	250 Kbps
	FireFly	ATmega1281 (8 bit) 8 MHz	8 KB	128 KB	CC2420	250 Kbps
Intermediate-class	Tmote Sky	MSP430 F1611 (16 bit) 8 MHz	10 KB	48 KB	CC2420	250 Kbps
	TelosB	Tl MSP430 (16 bit) 8 MHz	10 KB	1 MB	CC2420	250 Kbps
PDA-class	lmote2	PXA271 XScale (32 bit) 13–416 MHz	256 KB + 32MB SDRAM	32 MB	CC2420	250 Kbps
	Stargate	PXA255 XScale (32 bit) 400 MHz	64 MB	32 MB	CC2420 Bluetooth IEEE 802.1l	250 Kbps 1–3 Mbps 1–11 Mbps

**Table 5.** WMSN Camera Motes Features and Specifications.

Platform	Processor	Memory		Camera & Resolution	Radio	Power consumption
		RAM	Flash			
Cyclops [106]	8-bit ATMEl ATmega128L MCU + CPLD	64 KB	512 KB	Agilent compact CIF CMOS ADCM-1700 128×128 @ 10fps	Interfaced with Mica2 or Micaz IEEE 802.15.4	110 mW – 0.76 mW
Imote2 + Cam [102] [103]	32-bit PXA271 XScale processor (Imote2)	256 KB (Imote2)	32 MB (Imote2)	IMB400 camera OmniVision OV7649 640×480@30 fps	Integrated CC2420 IEEE 802.15.4	322 mW – 1.8 mW
FireFly Mosaic [107]	60MHz 32-bit LPC2106 ARM7TDMI MCU	64 KB	128 KB	CMUCam3 352×288 @ 50 fps	Interfaced with FireFly mote IEEE 802.15.4	572.3 mW – 0.29 mW
eCam [105]	OV 528 serial-bridge controller JPEG compression only	4 KB (Eco)	-	CoMedia C328–7640 (includes OV7640) 640×480 @ 30 fps	Interfaced with Eco wireless mote nRF24El radio RF 2.4 GHz 1Mbps	70 mA at 3.3V
MeshEye [110]	55 MHz 32-bit ARM7TDMI based on ATMEL AT91SAM7S	64 KB	256 KB	Agilent ADNS-3060 30×30 Agilent ADCM-2700 640×480 @ 10 fps	Integrated CC2420 IEEE 802.15.4	175.9 mW – 1.78 mW
Panoptes [100]	400 MHz 32-bit PXA255 XScale CPU (Stargate)	64 MB (Stargate)	32 MB (Stargate)	Logitech 3000 USB Camera 160×120 @ 30 fps 640×480 @ 13 fps	PCMCIA IEEE 802.11 wireless card	5.3 W – 58 mW
Wica [108]	84 MHz Xetal II SIM D+8051 ATMEL MCU	1.79 MB + 128 KB DP RAM	64 KB	VGA color camera 640×480 @ 30 fps	Aquis Grain ZigBee IEEE 802.15.4	600 mW max
MicrelEye [15]	8-bit ATMEL FPSLIC (includes 40kG FPGA)	36 KB + 1 MB external SRAM	-	Omnivision OV7640 320×240 @ 15 fps	LMX9820A Bluetooth 230.4 Kbps	500 mW max
WiSN [104]	48 MHz 32-bit ARM7TDMI based on ATMEL AT91SAM7S	64 KB	256 KB	Agilent ADCM-1670 352×288 @ 15 fps Agilent ADNS-3060 30×30 @ 100 fps	Integrated CC2420 IEEE 802.15.4	110 mA – 3 mA at 3.3V
CITRIC [95]	624 MHz 32-bit Intel XScale PXA270 CPU	64 MB	16 MB	Omnivision OV9655 1280×1024 @ 15 fps 640×480 @ 30 fps	Interfaced with T mote Sky mote IEEE 802.15.4	1 W max
Fox + Cam [14]	100 MHz LX416 Fox board	16 MB	4 MB	Labtec Webcam bro QuickCam Zoom 640×480	USB Bluetooth IEEE 802.15 100 m	1.5 W at 5 V
XYZ + Cam [102]	58MHz 32-bit ARM7TDMI based on OKIML67Q5002 (XYZ)	32 KB (XYZ)	256 KB + 2 MB on board (XYZ)	Omnivision OV7649 640×480 320×240 @ 4.1 fps	Integrated CC2420 IEEE 802.15.4 (XYZ)	238.6 mW – 2.2 mW

**Table 6.** WMSN Testbeds Features.

	**Testbed Name**	**Camera & Resolution**	**Wireless Mote**	**Additional Features**
**Software - Testbeds**	WiSNAP	Includes device library of: Agilent ADCM-1670	Includes device library of: Chipcon CC2420DB IEEE 802.15.4	- Matlab-based testbed - Open source APIs - Multimedia processing primitives
	AER Emulator	OmniVision OV7649 640×480 @ 30 fps 320×240 @ 60 fps	XYZ, Imote2 IEEE 802.15.4	- VisualC++ based testbed - AE recognition
**Hardware - Testbeds**	Meerkat	Logitech QuickCam Pro 4000 640×480	Stargate IEEE 802.11b	- Energy efficient - Event detection
	SenseEye	Cyclops, CMUCam3, PTZ Sony SNC-RZ30N Different resolutions	Mica2 IEEE 802.15.4 Stargate IEEE 802.11	- Multi-level resolution - surveillance application
	IrisNet	Logitech QuickCam Pro 4000 640×480	Stargate	- Internet-like queries - Scalable
	Explorebots	XI0 Cam2 320×240	Mica2 IEEE 802.15.4	- Mobile robot - electronic compass and ranging devices for navigation
	Mobile Emulab	Overhead Hitachi KP-D20A 768×494	Mica2 IEEE 802.15.4 Stargate IEEE 802.11b	- Mobile robot - Evaluate mobility-related network protocols
	WMSN-testbed	Logitech QuickCam Pro 4000 640×480 176 × 144 @ 15 fps	Micaz IEEE 802.15.4 Stargate IEEE 802.11b	- Mobile robot - Multi-level resolution
